# Development and Validation of the Composite Renal–Inflammatory–Nutritional–Metabolic Index for Predicting 28-Day Mortality in Critically Ill Patients

**DOI:** 10.3390/diagnostics16152443

**Published:** 2026-08-03

**Authors:** Temel Kayan, Fatih Segmen, Cihangir Doğu, Esra Yakışık Aktekin, Firdevs Tuğba Bozkurt Biçer, Şerife Bektaş, Recep Dokuyucu, Semih Aydemir

**Affiliations:** 1 Department of Intensive Care Unit, Ankara City Hospital, University of Health Sciences, 06800 Ankara, Türkiye; temel_kayan@hotmail.com (T.K.); drsegmen@gmail.com (F.S.); cihangirdogu@gmail.com (C.D.); pavlounmouse@hotmail.com (E.Y.A.); serifebektas@gmail.com (Ş.B.); 2 Department of Intensive Care Unit, School of Medicine, Yıldırım Beyazıt University, 06800 Ankara, Türkiye; drtugbabicu@gmail.com; 3 Department of Physiology, Medical Specialty Training Center (TUSMER), 06420 Ankara, Türkiye; drecepfatih@gmail.com; 4 Department of Critical Care, Yenimahalle Training and Research Hospital, Yıldırım Beyazıt University, 06010 Ankara, Türkiye

**Keywords:** RIN-IM Score, intensive care unit, mortality prediction, systemic inflammation, albumin, creatinine, TyG, SII, PIV

## Abstract

**Background/Objectives**: This study aimed to develop and internally validate the RIN-IM model, which integrates routinely measured biomarkers reflecting renal dysfunction, systemic immune-inflammatory activation, albumin-related alterations during acute illness, and metabolic stress, for prediction of 28-day mortality in critically ill adults. **Methods**: This retrospective cohort study included 1000 adults admitted to the intensive care unit (ICU) between January 2020 and June 2025. The first available demographic, clinical, and laboratory measurements obtained within 24 h of ICU admission were analyzed. The five prespecified RIN-IM predictors—serum creatinine, serum albumin, SII, PIV, and the TyG index—were initially evaluated as continuous variables; SII and PIV were natural-log-transformed, and potential nonlinear associations were examined using restricted cubic splines. Ridge-penalized logistic regression was used to address coefficient instability arising from correlation between SII and PIV. After evaluation of the primary continuous model, a simplified point-based score was derived by assigning 0–2 points to clinically ordered categories for each component, producing a total score of 0–10. Scores were categorized as low- (0–3), intermediate- (4–6), or high-risk (7–10). The primary outcome was 28-day all-cause mortality. Model performance was assessed using discrimination, calibration, Brier score, formal paired AUC comparisons, incremental-value testing, and decision-curve analysis. The complete development procedure was internally validated using 1000 bootstrap resamples. **Results**: Overall, 300 of 1000 patients (30.0%) died within 28 days. Mortality increased across the RIN-IM risk categories, from 7.6% (32/420) in the low-risk group to 27.2% (98/360) in the intermediate-risk group and 77.3% (170/220) in the high-risk group (*p* < 0.001). After adjustment for the clinical reference variables, each one-point increase in the simplified RIN-IM Score was associated with higher odds of 28-day mortality (adjusted OR, 1.68; 95% CI, 1.54–1.84; *p* < 0.001). The primary continuous model had an apparent AUC of 0.907 (95% CI, 0.885–0.929) and an optimism-corrected AUC of 0.898. The corresponding values for the simplified score were 0.890 (95% CI, 0.861–0.919) and 0.878, respectively. The apparent difference between the continuous and simplified models was 0.017 (95% CI, 0.007–0.028; *p* = 0.001). The simplified RIN-IM Score had higher discrimination than APACHE II (ΔAUC, 0.070; 95% CI, 0.038–0.102; *p* < 0.001) and SOFA (ΔAUC, 0.090; 95% CI, 0.057–0.123; *p* < 0.001). Adding RIN-IM to the clinical reference model increased the apparent AUC from 0.821 to 0.923 (ΔAUC, 0.102; 95% CI, 0.078–0.126; *p* < 0.001) and improved the Brier score from 0.160 to 0.112. **Conclusions**: In this single-center cohort, the RIN-IM model showed good discrimination and calibration for predicting 28-day mortality. The simplified score retained most of the predictive information of the continuous model and may complement established clinical severity scores using routinely available early laboratory measurements. External validation and clinical-impact studies are required before routine implementation.

## 1. Introduction

Critical illness triggers a cascade of metabolic, inflammatory, and immunological disturbances that collectively shape patient outcomes in the intensive care unit (ICU). Despite decades of research, early and accurate prediction of short-term mortality remains challenging in heterogeneous ICU populations. Established severity scores such as APACHE II and SOFA provide clinically valuable and widely accepted prognostic information. APACHE II incorporates numerous physiological, age-related, and chronic-health variables, whereas SOFA summarizes dysfunction across six organ systems. Their calculation is commonly supported by electronic systems, and neither score should be regarded as unsuitable for routine practice. Nevertheless, they require clinical and physiological information collected over time, and their predictive performance can vary across diagnostic groups [1,2,3,4,5,6,7,8]. As ICU patient numbers increase and resource demands grow, there is a rising need for prognostic tools that are simpler, faster, and grounded in readily available laboratory biomarkers.

Inflammation-based indices have gained considerable attention as predictors of mortality in acute and chronic disease. The systemic immune-inflammation index (SII) and the pan-immune-inflammation value (PIV), derived from neutrophil, lymphocyte, monocyte, and platelet counts, have shown strong associations with mortality in conditions ranging from sepsis to COVID-19 and malignancy [9,10,11,12,13,14,15,16]. These indices capture the complex interplay between innate immune activation and lymphocyte suppression, both fundamental features of critical illness.

Beyond inflammation, metabolic derangements play a central role in ICU outcomes. The triglyceride-glucose index (TyG) is widely recognized as a surrogate marker for insulin resistance, a key driver of endothelial dysfunction, impaired immune response, and multiorgan failure in critically ill patients. Several clinical studies and meta-analyses have confirmed the association between TyG and major adverse cardiovascular events, mortality, and cardiometabolic instability [17,18,19,20,21]. Similarly, the Atherogenic Index of Plasma (AIP) reflects underlying dyslipidemia and oxidative stress, both of which are frequently amplified in severe systemic illness [22,23,24,25,26,27].

Serum albumin is a nonspecific biomarker influenced by systemic inflammation, capillary leakage, hemodilution, hepatic synthetic function, redistribution, and nutritional status; therefore, low albumin concentrations in critically ill patients should not be interpreted as a specific measure of malnutrition. Hypoalbuminemia reflects a combination of inflammation, capillary leak, and malnutrition, and is consistently associated with higher mortality, prolonged mechanical ventilation, and acute kidney injury [28,29,30,31,32,33,34,35]. Serum creatinine provides a routinely available measure of renal dysfunction, whereas serum albumin reflects inflammatory burden, capillary leakage, and albumin-related alterations during acute illness [36,37]. These biomarkers were therefore evaluated separately during development of the RIN-IM Score.

Several laboratory-derived indices have been investigated for prognostic assessment in critically ill patients. These include the Glasgow Prognostic Score and modified Glasgow Prognostic Score, which combine CRP and albumin; the Prognostic Nutritional Index, based on albumin and lymphocyte count; inflammatory indices such as the neutrophil-to-lymphocyte ratio, SII, and PIV; and metabolic or organ-dysfunction markers such as the TyG index, CRP-to-albumin ratio, and lactate-to-albumin ratio [38,39,40,41,42]. However, these indices generally represent one or two pathophysiological domains, and few readily calculable tools simultaneously integrate renal dysfunction, systemic immune-inflammatory activation, albumin-related alterations during acute illness, and metabolic stress.

Although RIN-IM incorporates five biomarker domains, it requires only routinely available complete blood count and biochemical measurements obtained during the early ICU admission period. SII, PIV, and the TyG index can be calculated automatically from these laboratory results within the laboratory information system, electronic health record, or a simple electronic calculator. After calculation of the component indices, each variable is assigned 0, 1, or 2 points according to Table 1, and the points are summed without coefficient-based weighting. Thus, the simplified score does not require collection of numerous physiological observations, treatment variables, or chronic-health information. The primary continuous model was developed for prognostic evaluation, whereas the simplified score was intended as a pragmatic, calculator-assisted bedside representation.

We hypothesized that the continuous RIN-IM model would provide prognostic information for 28-day mortality beyond a conventional clinical reference model and that the simplified 0–10 point score would retain most of the predictive information of the continuous model. Accordingly, this study developed and internally validated both specifications and evaluated their discrimination, calibration, incremental prognostic value, and clinical net benefit.

## 2. Materials and Methods

### 2.1. Study Design and Setting

This study was designed as a retrospective, observational cohort study. The study was conducted at Ankara Etlik City Hospital, whereas ethical approval for the study protocol was granted by the Ethics Committee of Ankara Bilkent City Hospital (Approval No: TABED 2-26-1821; 7 January 2026). All procedures were conducted in accordance with the ethical standards of the institutional and national research committee and with the 1964 Helsinki Declaration and its later amendments.

This retrospective cohort study was conducted in the Intensive Care Unit (ICU) of Ankara Etlik City Hospital, a tertiary-level academic medical center providing multidisciplinary critical care services. The study covered a 5.5-year period from January 2020 to June 2025, during which all adult ICU admissions were screened for eligibility. The study protocol was developed based on the predefined research framework for evaluating biomarker-derived risk indices in critical illness.

The primary reason for ICU admission was determined from the ICU admission note and discharge coding and was classified into mutually exclusive diagnostic categories: sepsis or septic shock; acute respiratory failure unrelated to COVID-19; COVID-19-related critical illness; acute neurological disease; cardiovascular disease; trauma; postoperative admission; gastrointestinal or hepatic disease; renal, metabolic, or toxicological disease; and other diagnoses. When more than one condition was present, the condition judged to be the immediate indication for ICU admission was designated as the primary diagnosis. Diagnostic classification was independently reviewed by two investigators, and disagreements were resolved by consensus. Admissions were also classified as medical or surgical. Medical admissions included patients admitted without a preceding operative or trauma-related indication. Surgical admissions included scheduled or emergency postoperative admissions and trauma patients managed primarily by a surgical service.

**Figure 1:**
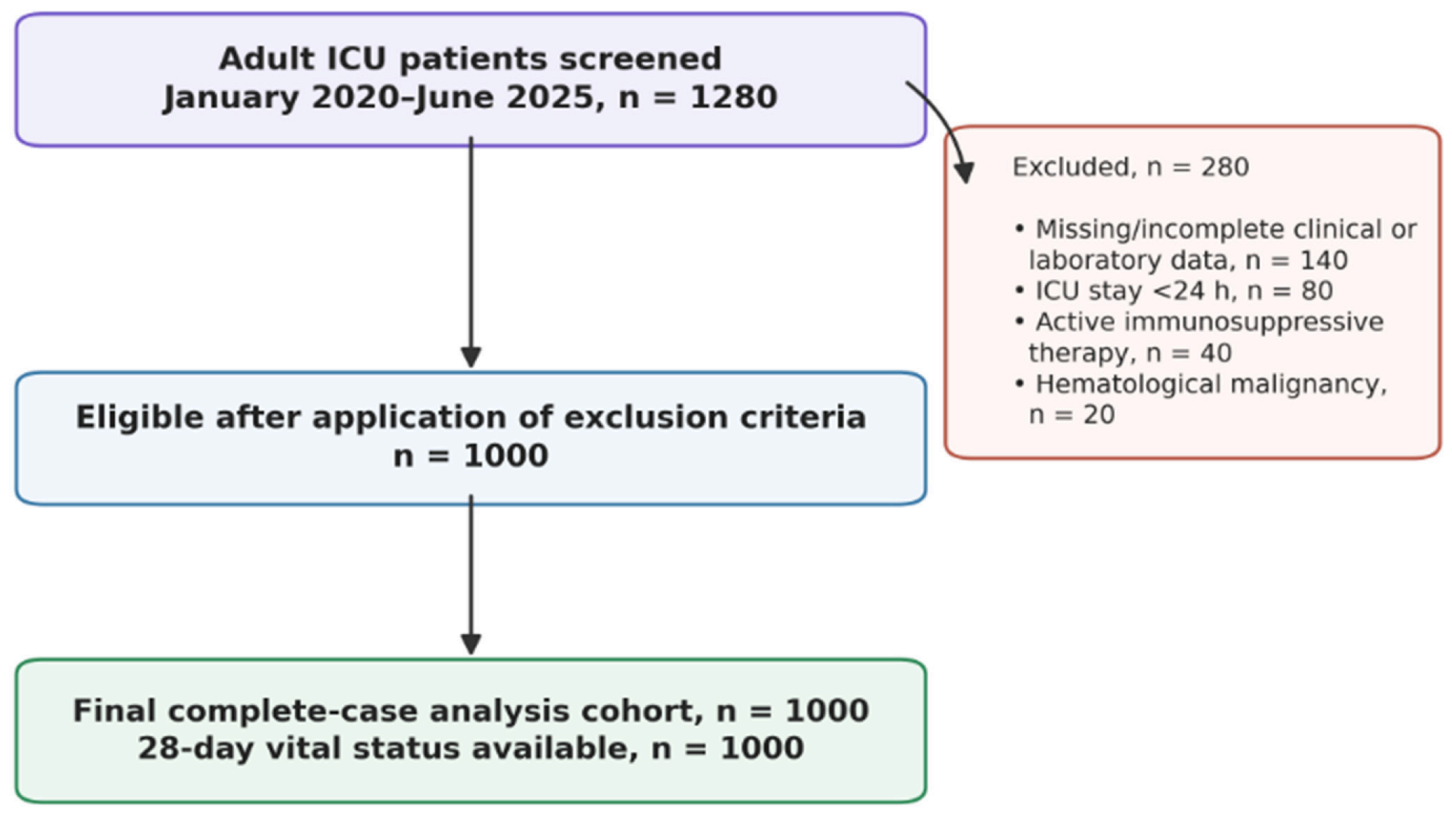
Flow diagram of patient screening, exclusions, and the final analytical cohort.

All adult patients admitted to the intensive care unit between January 2020 and June 2025 were screened for eligibility. Individuals were included if they were 18 years of age or older, had complete laboratory results available within the first 24 h of ICU admission, and had documented 28-day survival or mortality outcomes. Patients were excluded if they had missing or incomplete laboratory or clinical data, a known diagnosis of hematological malignancy, or were receiving active immunosuppressive therapy such as corticosteroids at a dose of ≥20 mg/day or biological agents at the time of admission. Additional exclusions included ICU stays of less than 24 h and repeated ICU admissions during the same hospitalization, in which case only the first admission was considered for analysis. A flow diagram summarizing the screening and selection process is presented in Figure 1.

### 2.2. Data Collection

Data for all eligible patients were retrieved retrospectively from the hospital’s electronic medical record system using a standardized collection protocol. Two independent investigators extracted demographic information, including age, sex, body mass index, and major comorbidities such as hypertension, diabetes mellitus, coronary artery disease, and chronic kidney disease. Clinical variables related to the severity and course of critical illness—such as primary reason for ICU admission, APACHE II and SOFA scores at presentation, need for mechanical ventilation, vasopressor use within the first 24 h, ICU length of stay, and 28-day mortality—were recorded in detail. ICU admission was defined as time zero. APACHE II and admission SOFA scores were calculated once using the worst eligible physiological and laboratory values recorded from ICU admission through 24 h. RIN-IM components were derived from the first available blood samples obtained after ICU admission and no later than 24 h; all components were obtained from the same blood draw whenever available. Thus, the scoring windows overlapped, although APACHE II and SOFA reflected the worst values during the first 24 h rather than a single admission measurement. Comparability was additionally examined in the sensitivity analysis restricted to patients whose complete RIN-IM sampling was performed within 2 h of admission. During the study period, CRP and admission triglyceride measurements were included in the institutional ICU admission laboratory panel and were obtained as part of routine clinical care rather than specifically for this study. The complete cholesterol profile was collected only when clinically available and was used for exploratory calculation of AIP. Neither CRP, total cholesterol, HDL cholesterol, LDL cholesterol, nor AIP was required for calculation of the RIN-IM Score or for inclusion in the primary model. Laboratory parameters obtained during the first 24 h of ICU admission were also collected, including complete blood count components (neutrophils, lymphocytes, monocytes, and platelets), renal function tests (creatinine and urea), serum albumin, lipid profile (total cholesterol, triglycerides, HDL-cholesterol, LDL-cholesterol), admission glucose, and C-reactive protein levels. Complete blood counts were measured using a Sysmex XN-1000 automated hematology analyzer (Sysmex Corporation, Kobe, Japan). Leukocyte differential counts were determined by fluorescence flow cytometry, whereas platelet counts were measured using the impedance method. Serum creatinine, albumin, admission glucose, triglycerides, and C-reactive protein (CRP) were analyzed using a cobas c 702 automated biochemistry analyzer (Roche Diagnostics GmbH, Mannheim, Germany). Serum creatinine was measured using an isotope-dilution mass-spectrometry-traceable compensated kinetic Jaffé method, albumin using the bromocresol green method, glucose using the hexokinase method, triglycerides using an enzymatic colorimetric method, and CRP using particle-enhanced immunoturbidimetry. Calibration was performed using manufacturer-specified calibrators at scheduled intervals, following reagent-lot changes, and whenever indicated by internal quality-control results. Results were accepted only when quality-control measurements were within the laboratory’s predefined limits. The manufacturer-specified analytical measurement ranges were 0.2–6.0 g/dL for albumin, 2–750 mg/dL for glucose, and 8.85–885 mg/dL for triglycerides. Extracted data were checked for consistency and completeness by a third investigator, and discrepancies were resolved by consensus to ensure accuracy and reliability prior to statistical analysis.

ICU admission time was defined as time zero. For each RIN-IM component, the interval between ICU admission and specimen collection was calculated using the recorded specimen collection timestamp rather than the laboratory result-validation time. The first available value within 24 h was used in the primary analysis. For each patient, the RIN-IM sampling-completion time was defined as the latest collection time among the specimens required to calculate serum creatinine, serum albumin, SII, PIV, and the TyG index. Because complete blood count and serum biochemical measurements require different collection tubes, “same blood draw” was operationally defined as collection during the same phlebotomy episode, with all required specimen timestamps occurring within 15 min. The timing of specimen collection relative to documented initiation of vasopressors, invasive mechanical ventilation, intravenous glucose or insulin, corticosteroids, enteral or parenteral nutrition, blood-product transfusion, and renal replacement therapy was also assessed when reliable treatment timestamps were available. Interventions initiated before ICU admission or lacking a reliable timestamp were classified as having indeterminate temporal status.

Several biomarker-derived indices were computed using standard formulas established in prior clinical research to quantify renal function, inflammatory burden, nutritional status, and metabolic dysregulation. Separately from development of the RIN-IM Score, a secondary exploratory serum albumin-to-creatinine measure was calculated as serum albumin (g/dL) divided by serum creatinine (mg/dL) and was reported as g/mg: sAlb/Cr = serum albumin (g/dL)/serum creatinine (mg/dL). The notation sAlb/Cr was used to distinguish this serum-derived measure from the urinary albumin-to-creatinine ratio, conventionally denoted as uACR. Urinary albumin was not measured in the present study. The serum-derived ratio was evaluated because previous retrospective studies reported associations between lower serum albumin-to-creatinine ratios and mortality in critically ill populations [36,37]. However, sAlb/Cr was not a component of the RIN-IM Score and was not entered into multivariable models together with serum albumin and serum creatinine. Pre-existing chronic kidney disease (CKD) was identified only when a CKD diagnosis had been documented in the medical record before the index ICU admission. CKD was not inferred from a single admission creatinine measurement. Stable preadmission serum creatinine values, longitudinal eGFR measurements, and information required to classify CKD by GFR category were not uniformly available; therefore, CKD stage could not be reliably determined. Similarly, serial serum creatinine measurements and complete hourly urine-output data were insufficient for consistent application of KDIGO AKI criteria. Admission creatinine was consequently treated only as a prognostic biomarker and not as evidence of AKI, CKD, or AKI superimposed on CKD. The systemic immune-inflammation index (SII) was calculated as platelet count × neutrophil count/lymphocyte count, with all blood-cell counts expressed as ×10^9^/L. The pan-immune-inflammation value (PIV) was calculated as neutrophil count × platelet count × monocyte count/lymphocyte count, likewise using cell counts expressed as ×10^9^/L. SII and PIV were reported in conventional arbitrary units. The atherogenic index of plasma (AIP) was calculated as log_10_[triglycerides (mmol/L)/HDL cholesterol (mmol/L)]. Metabolic indices included the Atherogenic Index of Plasma (AIP), calculated as log_10_[triglycerides (mmol/L)/HDL cholesterol (mmol/L)], and the admission-based triglyceride–glucose (TyG) index, calculated as ln{[triglycerides (mg/dL) × admission glucose (mg/dL)]/2}, where ln denotes the natural logarithm. Triglyceride and HDL-cholesterol concentrations reported in mg/dL were converted to mmol/L before calculation of AIP. Because fasting status, sampling from the same phlebotomy episode, and measurement before relevant therapeutic interventions could not be uniformly verified, the admission-based TyG index was interpreted as a marker of early metabolic dysregulation during critical illness rather than as a validated measure of chronic insulin resistance.

### 2.3. Development and Internal Validation of the Composite Renal–Inflammatory–Nutritional–Metabolic Index for Predicting 28-Day Mortality in Critically Ill Patients

**Figure 2:**
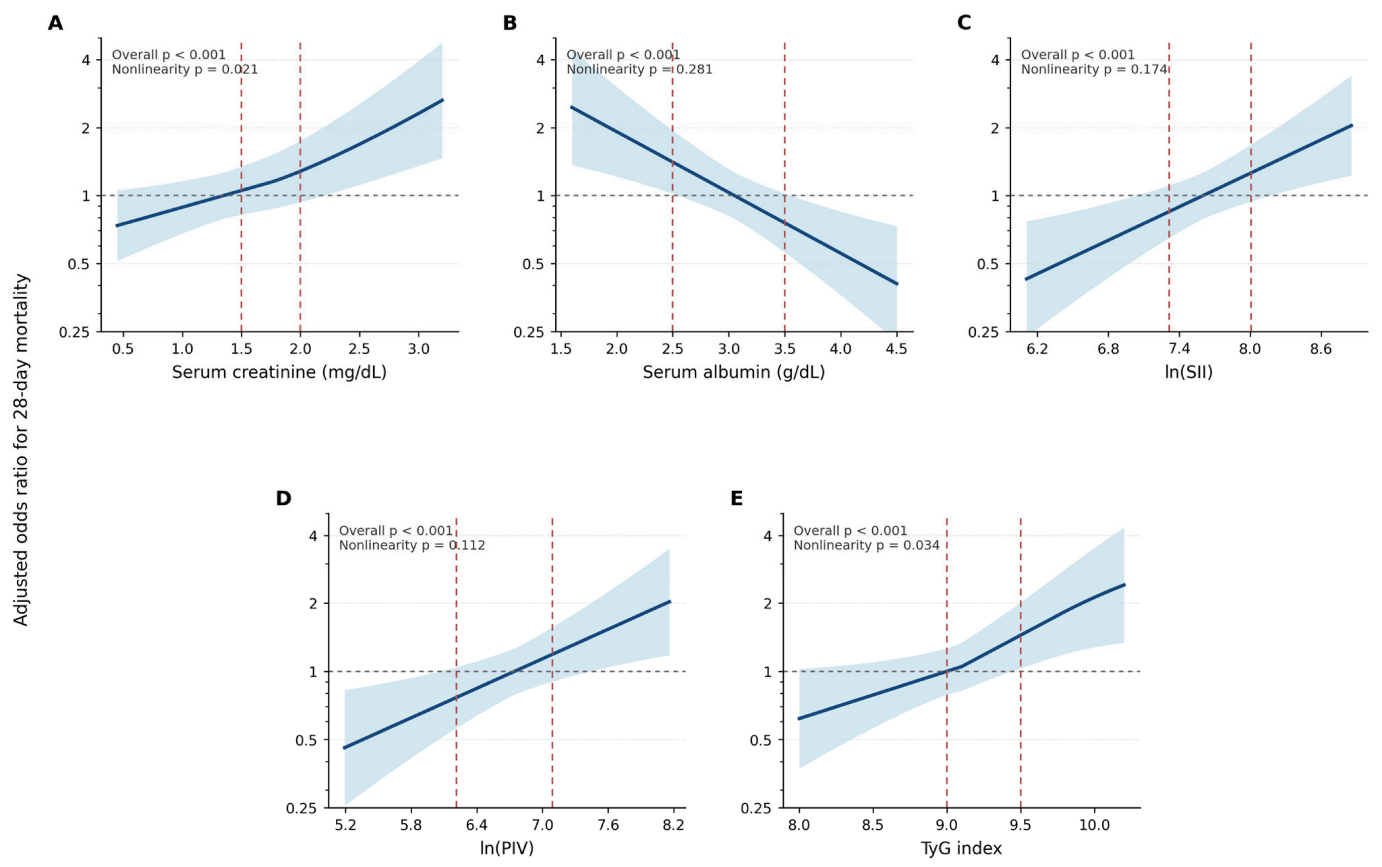
Restricted cubic spline associations of serum creatinine (**A**), serum albumin (**B**), natural-log-transformed SII (**C**), natural-log-transformed PIV (**D**), and the TyG index (**E**) with the adjusted odds of 28-day mortality. Solid lines indicate adjusted odds ratios, and shaded areas indicate 95% confidence intervals. The median value of each predictor was used as the reference. Vertical dashed lines indicate the thresholds subsequently used to derive the simplified point-based RIN-IM Score. Each predictor was mutually adjusted for the other continuous RIN-IM components. SII, systemic immune-inflammation index; PIV, pan-immune-inflammation value; TyG, triglyceride-glucose index. The functional forms, likelihood-ratio test results, and adjusted interquartile-range contrasts are reported in Supplementary Table S1.

The five-candidate RIN-IM predictors—serum creatinine, serum albumin, SII, PIV, and the TyG index—were prespecified before outcome modeling on the basis of their representation of the four RIN-IM domains, prior prognostic evidence, and routine availability during early ICU admission. Predictor inclusion was not determined by univariable statistical significance. All predictors were initially evaluated as continuous variables. Because of their right-skewed distributions, SII and PIV were natural-log-transformed before model development. The functional relationship between each predictor and the log odds of 28-day mortality was examined using restricted cubic splines with three knots located at the 10th, 50th, and 90th percentiles. Departure from linearity was evaluated using likelihood-ratio tests comparing models containing linear terms with those containing spline terms. Categorization was performed only after evaluation of the continuous model because premature categorization may cause information loss, reduced predictive performance, and unstable sample-dependent thresholds [38]. The component thresholds were not selected using single-predictor ROC analyses or the Youden index. Albumin categories were prespecified using clinically recognized hypoalbuminemia strata [28,29,30,31,32,33,34,35]. For creatinine, SII, PIV, and TyG, cohort-derived candidate transition points were identified from the continuous predictor distributions and fitted spline functions and were subsequently rounded to clinically interpretable values. The selected thresholds were required to produce ordered categories with monotonic mortality gradients. Their locations relative to the continuous risk functions are shown in Figure 2, while the functional forms, overall association tests, nonlinearity tests, and adjusted effect estimates are reported in Supplementary Table S1.

The point-based RIN-IM Score was subsequently derived as a simplified representation of the continuous model for potential bedside use. The renal component of the RIN-IM Score was based on the first available serum creatinine concentration obtained after ICU admission and within 24 h. Admission creatinine was evaluated as a continuous predictor in the primary model. For derivation of the simplified score, the cohort-derived thresholds were clinically rounded to <1.50, 1.50–1.99, and ≥2.00 mg/dL and assigned 0, 1, and 2 points, respectively. These absolute thresholds were not based on KDIGO acute kidney injury stages and were not intended to diagnose or classify AKI. Because published SII and PIV thresholds vary considerably across ICU populations, their thresholds were treated as cohort-derived rather than universally established clinical cut-offs [39,40]. Similarly, previous ICU studies have demonstrated nonlinear associations between the TyG index and mortality, with transition points ranging from approximately 8.82 to 9.84, supporting the use of clinically rounded thresholds in the simplified score [41,42]. The complete development procedure—including predictor transformation, assessment of nonlinearity, threshold selection, and score derivation—was repeated across 1000 bootstrap samples to estimate optimism-corrected performance [43].

After confirmation of monotonic risk gradients and broadly comparable standardized effect estimates, each predictor was assigned 0, 1, or 2 points according to the thresholds presented in Table 1. The 0-, 1-, and 2-point assignments were not generated by directly rounding the regression coefficients; they represented a pragmatic equal-point simplification based on the ordered risk gradients and broadly comparable standardized associations of the five components. Component points were summed without additional weighting according to the following equation: RIN-IM Score = creatinine points + SII points + PIV points + albumin points + TyG points, yielding a total score ranging from 0 to 10. Total scores were classified as low risk (0–3 points), intermediate risk (4–6 points), or high risk (7–10 points). These categories were defined pragmatically by dividing the possible 0–10 score range into three approximately equal-width intervals. They were not selected using ROC analysis, the Youden index, or prespecified clinical-action thresholds. The subsequent mortality rates across these categories were evaluated to confirm an ordered risk gradient. Accordingly, the categories should be interpreted as descriptive risk strata requiring external validation. CRP, admission glucose, triglycerides, AIP, and the serum albumin-to-creatinine ratio were evaluated separately and were not independently scored. Admission glucose and triglyceride concentrations contributed to the RIN-IM Score only through calculation of the TyG index. Because several thresholds were derived from the present cohort, they should not be interpreted as externally validated clinical cut-offs. The complete scoring algorithm is provided in Table 1.

Calculation of the RIN-IM Score did not require calculation of AIP, sAlb/Cr, APACHE II, or SOFA. SII and PIV were generated from the same complete blood count, and the TyG index was generated from admission triglyceride and glucose measurements. The resulting five component points were then summed without additional weighting. APACHE II and SOFA were calculated only as reference comparators and were not components of RIN-IM. In practice, the three derived indices can be generated automatically by the laboratory information system or electronic health record.

### 2.4. Outcomes

The sole prespecified outcome was 28-day all-cause mortality, defined as death from any cause within 28 days after ICU admission. Twenty-eight-day vital status was determined using [the hospital electronic follow-up system/the national death-notification system] and was available for all included patients. No patient was lost to follow-up before day 28. Mechanical ventilation and vasopressor use during the first 24 h were recorded as early clinical characteristics and potential adjustment variables rather than as study outcomes. ICU length of stay was considered a descriptive post-admission variable and was not included as a secondary endpoint or predictor in the mortality models.

### 2.5. Statistical Analysis

All statistical analyses were performed using IBM SPSS Statistics version 27.0 (IBM Corp., Armonk, NY, USA) and R software (version 4.3.0; R Foundation for Statistical Computing, Vienna, Austria). Distributions of continuous variables were assessed using histograms, Q–Q plots, and the Shapiro–Wilk test. Normally distributed variables were presented as mean ± standard deviation, and non-normally distributed variables as median and interquartile range. Categorical variables were summarized as frequencies and percentages. Between-group comparisons were performed using the independent-samples Student’s t-test or Mann–Whitney U test, as appropriate. Categorical variables were compared using the chi-square test or Fisher’s exact test. Analyses were restricted to patients with complete data for the five prespecified predictors and 28-day mortality; no missing-data imputation was performed.

The primary outcome was 28-day all-cause mortality. Associations with mortality were initially examined using univariable logistic regression and reported as odds ratios with 95% confidence intervals. Univariable statistical significance was not used as a criterion for predictor inclusion. The primary continuous RIN-IM model included serum creatinine, serum albumin, natural-log-transformed SII, natural-log-transformed PIV, and the admission-based TyG index. Potential non-linear associations were examined using restricted cubic splines with three knots located at the 10th, 50th, and 90th percentiles. Departure from linearity was evaluated using likelihood-ratio tests comparing models containing linear terms with models containing the corresponding spline terms.

To avoid mathematical redundancy, the total RIN-IM Score and its constituent biomarkers were not entered into the same multivariable model. Four prespecified logistic regression models were evaluated: Model A was the clinical reference model and included age, sex, hypertension, diabetes mellitus, coronary artery disease, chronic kidney disease, and SOFA; Model B was the biomarker-component model and included serum creatinine, serum albumin, ln(SII), ln(PIV), and the TyG index; Model C included the simplified RIN-IM Score alone; and Model D combined the variables in Model A with the total RIN-IM Score. The score was modeled per one-point increase. Continuous biomarker associations were expressed using contrasts between the 75th and 25th percentiles. For predictors modeled non-linearly, these contrasts were calculated from the fitted restricted cubic spline functions rather than from a single linear coefficient.

SOFA was used in the primary clinical reference model. APACHE II and SOFA were not entered simultaneously because they represent overlapping aspects of acute physiological derangement and organ dysfunction. As a sensitivity analysis, parsimonious APACHE II-based models containing sex and APACHE II, with and without the RIN-IM Score, were evaluated separately. Exact age was not re-entered into these models because age and chronic health status are incorporated into the APACHE II total score. Pairwise correlations were assessed using Spearman correlation coefficients. Model-specific multicollinearity was evaluated using variance inflation factors and tolerance statistics. A variance inflation factor greater than 5 or a tolerance value below 0.20 was considered indicative of potentially important multicollinearity. Because SII and PIV share hematological components, coefficient stability was additionally examined using ridge-penalized logistic regression, with the penalty parameter selected by 10-fold cross-validation. Detailed collinearity diagnostics are presented in Supplementary Table S2.

Model discrimination was quantified using the area under the receiver operating characteristic curve with 95% confidence intervals. Pairwise comparisons of correlated AUCs were performed using the nonparametric paired DeLong method [44], and absolute AUC differences were reported with 95% confidence intervals and two-sided *p* values. Calibration was evaluated using the calibration intercept, calibration slope, Brier score, and graphical calibration plots. Performance of the continuous biomarker model was compared with that of the simplified point-based score to quantify the loss of predictive information resulting from categorization. Incremental prognostic value was evaluated by comparing Model A with the nested Model D using the likelihood-ratio test and changes in AUC, calibration intercept, calibration slope, and Brier score. Decision-curve analysis compared the clinical reference model, the RIN-IM Score-only model, and the clinical reference model plus the RIN-IM Score with the treat-all and treat-none strategies across threshold probabilities from 5% to 70% [44].

Internal validation was performed using 1000 bootstrap resamples. The complete model-development process—including predictor transformation, assessment of nonlinearity, threshold selection, and derivation of the simplified point score—was repeated within each bootstrap sample. Optimism was estimated by comparing performance in each bootstrap sample with performance obtained when the bootstrap-derived model was applied to the original dataset. Apparent and optimism-corrected estimates of discrimination, calibration, and Brier score were reported separately [43]. For the ridge-penalized sensitivity analysis, the penalty parameter was re-estimated by cross-validation within each bootstrap resample.

Logistic regression was retained as the primary modeling approach because the study evaluated the fixed-horizon probability of death by day 28 and complete 28-day vital status was available for all included patients. As a time-to-event sensitivity analysis, follow-up was calculated from ICU admission to death or day 28. Patients alive at day 28 were administratively censored at that time. Kaplan–Meier curves were generated for the low-, intermediate-, and high-risk RIN-IM groups and compared using the log-rank test. Cox proportional-hazards models were used to estimate hazard ratios with 95% confidence intervals for the continuous score and its risk categories. The proportional-hazards assumption was evaluated using scaled Schoenfeld residuals.

The time from ICU admission to completion of RIN-IM component sampling was summarized as median and interquartile range. The proportions of patients with all required specimens collected within 2 and 6 h were reported. The same phlebotomy episode was defined as collection of all required specimens within 15 min. A sensitivity analysis was performed among patients for whom all five components were available within 2 h of ICU admission. The prespecified thresholds were applied without re-estimation, and discrimination, calibration, and Brier score were recalculated. An additional sensitivity analysis was performed among patients whose specimens preceded all reliably timestamped major therapeutic interventions. Sampling-time analyses are presented in Supplementary Table S4.

Prespecified exploratory subgroup analyses were performed according to admission type, sepsis status, mutually exclusive primary admission diagnosis, and calendar period of ICU admission (2020–2021, 2022–2023, and 2024–June 2025). COVID-19-related critical illness was evaluated separately from other respiratory diagnoses. The continuous model and simplified score were applied without subgroup-specific refitting or threshold selection. Subgroup performance was summarized using AUC with 95% confidence intervals, calibration slope, calibration intercept, and Brier score. Heterogeneity in the association of the RIN-IM Score with mortality was examined using multiplicative interaction terms between the score and subgroup indicator. Performance estimates were not calculated for subgroups containing fewer than 20 deaths because of insufficient precision. These analyses were exploratory, and no adjustment for multiple comparisons was performed.

To explore temporal and diagnostic-spectrum heterogeneity, calendar period was added to Model D as a categorical variable, and COVID-19 diagnosis was included as an additional covariate. The adjusted association of the RIN-IM Score, optimism-corrected AUC, calibration measures, and Brier score were recalculated. The optimal binary threshold for the simplified RIN-IM Score was selected by maximizing the Youden index (sensitivity + specificity − 1) in the development cohort. The threshold of ≥6 points was data-derived and was not prespecified. Threshold selection was repeated within each bootstrap sample as part of internal validation. Accordingly, ≥6 should be regarded as an internally derived discriminatory threshold rather than an externally validated clinical decision cut-off. All statistical tests were two-sided, and *p* < 0.05 was considered statistically significant.

## 3. Results

### 3.1. Cohort Characteristics and Case Mix

A total of 1000 patients were included in the analysis, with 700 survivors and 300 non-survivors at 28 days. As shown in Table 2, non-survivors were substantially older than survivors (70.1 ± 14.3 vs. 60.1 ± 14.7 years), and the proportion of patients with major comorbidities—particularly hypertension, diabetes mellitus, coronary artery disease, and chronic kidney disease—was markedly higher in the non-survivor group. The cohort included 760 medical admissions (76.0%) and 240 surgical admissions, including trauma (24.0%). Sepsis or septic shock was the most frequent primary admission diagnosis, accounting for 310 patients (31.0%), followed by non-COVID-19 acute respiratory failure (*n* = 170, 17.0%), acute neurological disease (*n* = 120, 12.0%), cardiovascular disease (*n* = 95, 9.5%), and COVID-19-related critical illness (*n* = 90, 9.0%). Postoperative and trauma admissions accounted for 80 (8.0%) and 75 patients (7.5%), respectively. The distribution of primary admission diagnoses differed between survivors and non-survivors (omnibus *p* < 0.001). Sepsis or septic shock accounted for 46.7% of non-survivors and 24.3% of survivors. Non-survivors presented with a more severe clinical profile at admission, reflected by significantly higher APACHE II (25.2 ± 6.8 vs. 17.3 ± 6.2) and SOFA scores (10.6 ± 3.4 vs. 6.5 ± 2.8). They also required mechanical ventilation (76.7% vs. 41.4%) and vasopressor support (63.3% vs. 21.4%) more frequently during the first 24 h of ICU stay. In addition, non-survivors exhibited a longer median ICU length of stay compared with survivors (10 vs. 7 days) (Table 2).

### 3.2. Biomarker Comparisons

Inflammatory, nutritional, renal, and metabolic biomarkers differed between survivors and non-survivors (Table 3). Non-survivors exhibited significantly higher neutrophil counts (11.4 ± 4.8 vs. 8.2 ± 3.5 ×10^9^/L, *p* = 0.003) and lower lymphocyte counts (0.82 ± 0.34 vs. 1.25 ± 0.46 ×10^9^/L, *p* = 0.002). Accordingly, SII values were more than doubled in non-survivors compared with survivors (3800 (2500–5800) vs. 1600 (900–2500), *p* = 0.001), and PIVs were also substantially higher (1680 (980–2550) vs. 650 (320–980), *p* = 0.002). CRP levels demonstrated a similar pattern of heightened inflammation in the non-survivor group (148 (96–220) mg/L vs. 85 (40–132) mg/L, *p* = 0.021). Nutritional and renal parameters further differentiated the outcome groups. Non-survivors had lower serum albumin levels (2.6 ± 0.5 vs. 3.2 ± 0.6 g/dL; *p* = 0.004) and lower exploratory sAlb/Cr values (1.7 ± 0.9 vs. 2.8 ± 1.0 g/mg; *p* = 0.006), whereas serum creatinine was higher among non-survivors (1.92 ± 0.72 vs. 1.18 ± 0.46 mg/dL; *p* = 0.030). Metabolic derangements were also more prominent in the non-survivor group, with higher triglyceride levels (162 ± 68 vs. 136 ± 52 mg/dL, *p* = 0.048), increased admission glucose (182 ± 62 vs. 146 ± 45 mg/dL, *p* = 0.031), and a higher TyG (9.32 ± 0.58 vs. 8.85 ± 0.52, *p* = 0.012). Importantly, the composite RIN-IM Score showed a marked separation between groups, with non-survivors presenting more than double the score observed in survivors (7.2 ± 1.6 vs. 3.2 ± 1.4, *p* = 0.001) (Table 3).

### 3.3. Continuous Model Development and Derivation of the Simplified RIN-IM Score

In the primary model, serum creatinine, serum albumin, natural-log-transformed SII, natural-log-transformed PIV, and the TyG index were initially evaluated as continuous predictors. Restricted cubic spline analyses demonstrated evidence of nonlinearity for serum creatinine (*p* for nonlinearity = 0.021) and the TyG index (*p* for nonlinearity = 0.034). In contrast, the associations of serum albumin (*p* for nonlinearity = 0.281), ln-SII (*p* for nonlinearity = 0.174), and ln-PIV (*p* for nonlinearity = 0.112) with 28-day mortality were adequately represented by linear terms. The directions of the associations were consistent across the continuous model: mortality risk increased with higher creatinine, ln-SII, ln-PIV, and TyG values and with lower albumin concentrations.

The primary continuous model yielded an apparent AUC of 0.907 (95% CI: 0.885–0.929). After correction for optimism using 1000 bootstrap resamples, the AUC was 0.898 (bootstrap 95% CI: 0.875–0.918). The optimism-corrected calibration slope was 0.94, the calibration intercept was −0.02, and the Brier score was 0.123, indicating good overall agreement between predicted and observed 28-day mortality risks.

For derivation of the simplified RIN-IM Score, clinically rounded categories were applied only after evaluation of the continuous predictor–outcome relationships. Monotonic mortality gradients were observed across the defined categories, and the standardized effect estimates were broadly comparable, supporting assignment of 0, 1, and 2 points to the ordered categories. The resulting point-based RIN-IM Score yielded an apparent AUC of 0.890 (95% CI: 0.861–0.919) and a bootstrap optimism-corrected AUC of 0.878 (bootstrap 95% CI: 0.851–0.903). The apparent absolute difference in AUC between the continuous and simplified models was 0.017 (95% CI: 0.007–0.028; DeLong *p* = 0.001). The optimism-corrected calibration slope and intercept of the simplified score were 0.91 and −0.04, respectively, and its Brier score was 0.132 (Figure 2).

### 3.4. Multivariable Models and Incremental Prognostic Performance

Separate multivariable models for 28-day mortality are shown in Table 4. In the clinical reference model (Model A), age (OR per 10-year increase, 1.22; 95% CI, 1.10–1.35; *p* < 0.001), diabetes mellitus (OR, 1.32; 95% CI, 1.00–1.75; *p* = 0.049), chronic kidney disease (OR, 1.56; 95% CI, 1.11–2.20; *p* = 0.011), and SOFA score (OR per one-point increase, 1.24; 95% CI, 1.18–1.30; *p* < 0.001) were associated with 28-day mortality. In the biomarker-component model (Model B), the adjusted odds ratios comparing the 75th with the 25th percentile were 1.68 (95% CI, 1.39–2.04) for serum creatinine, 0.58 (95% CI, 0.48–0.70) for serum albumin, 1.54 (95% CI, 1.28–1.84) for ln(SII), 1.46 (95% CI, 1.22–1.75) for ln(PIV), and 1.61 (95% CI, 1.31–1.98) for the TyG index (all *p* < 0.001). In the score-only model (Model C), each one-point increase in the RIN-IM Score was associated with an OR of 1.88 (95% CI, 1.75–2.02; *p* < 0.001). After adjustment for the clinical reference variables (Model D), the corresponding OR was 1.68 (95% CI, 1.54–1.84; *p* < 0.001), while the adjusted OR for SOFA was 1.14 per point (95% CI, 1.08–1.21; *p* < 0.001). The apparent and optimism-corrected AUCs were 0.821 and 0.810 for Model A, 0.907 and 0.898 for Model B, 0.890 and 0.878 for Model C, and 0.923 and 0.912 for Model D, respectively. Addition of the RIN-IM Score to the clinical reference model resulted in an AUC difference of 0.102 (95% CI, 0.078–0.126; DeLong *p* < 0.001), with a likelihood-ratio χ^2^ of 166.4 (*p* < 0.001). The highest pairwise correlation was observed between ln(SII) and ln(PIV) (ρ = 0.81) (Table 4).

All model-specific VIFs were below 5, with a maximum VIF of 3.18 and a minimum tolerance of 0.314 (Supplementary Table S2).

Sensitivity analyses replacing SOFA with APACHE II are presented in Supplementary Table S3. In the APACHE II-based reference model, the adjusted OR for APACHE II was 1.12 per point (95% CI, 1.09–1.15; *p* < 0.001). After addition of the RIN-IM Score, the adjusted ORs were 1.07 per APACHE II point (95% CI, 1.04–1.10; *p* < 0.001) and 1.63 per RIN-IM point (95% CI, 1.49–1.79; *p* < 0.001). Addition of the RIN-IM Score increased the apparent AUC from 0.821 to 0.919 (ΔAUC, 0.098; 95% CI, 0.074–0.122; DeLong *p* < 0.001). The corresponding optimism-corrected AUCs were 0.812 and 0.908. The Brier score decreased from 0.159 to 0.115, and the nested-model likelihood-ratio test yielded χ^2^ = 155.7 (*p* < 0.001).

Although age, comorbidity burden, and conventional severity scores were strongly associated with mortality, the RIN-IM Score was evaluated as a complementary rather than replacement prognostic measure. Addition of RIN-IM to the SOFA-based clinical reference model increased the apparent AUC from 0.821 to 0.923 (ΔAUC, 0.102; 95% CI, 0.078–0.126; *p* < 0.001), reduced the Brier score from 0.160 to 0.112, and improved model fit (likelihood-ratio χ^2^ = 166.4; *p* < 0.001).

### 3.5. Risk Categories, Discrimination, and Calibration

Distribution of RIN-IM Score categories (low-, intermediate-, and high-risk) and associated mortality rates are shown in Table 5. Of the 420 patients in the low-risk category, 388 survived and 32 died (mortality, 7.6%). Among the 360 patients in the intermediate-risk category, 262 survived and 98 died (27.2%). Of the 220 patients in the high-risk category, 50 survived and 170 died (77.3%). Overall, the cohort comprised 700 survivors and 300 non-survivors (*N* = 1000) (Table 5).

**Figure 3:**
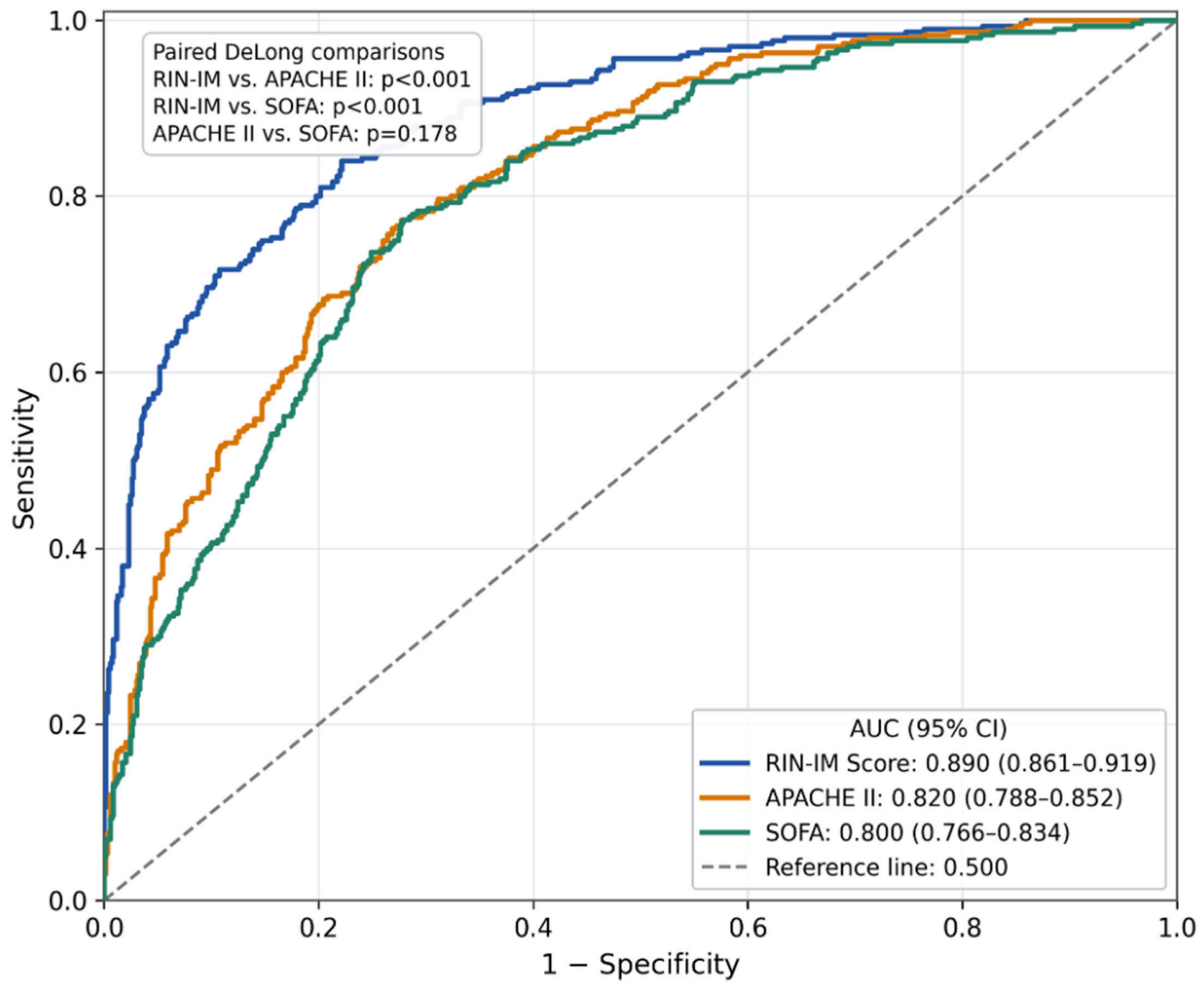
Receiver operating characteristic (ROC) curves comparing prognostic performance of the RIN-IM Score, APACHE II, and SOFA for predicting 28-day mortality.

In ROC curve analysis, the RIN-IM Score exhibited higher discrimination prognostic performance for 28-day mortality compared with conventional severity scores. The RIN-IM Score yielded the highest AUC (0.89, 95% CI 0.86–0.92), whereas the APACHE II and SOFA scores demonstrated AUC values of 0.82 (95% CI 0.78–0.86) and 0.80 (95% CI 0.76–0.84), respectively. Using an optimal cut-off of ≥6 points, the RIN-IM Score achieved a sensitivity of 84.0% and a specificity of 81.3%. Formal comparisons of the correlated ROC curves are presented in Table 6. The RIN-IM Score showed higher discrimination than APACHE II (ΔAUC, 0.070; 95% CI, 0.038–0.102; DeLong *p* < 0.001) and SOFA (ΔAUC, 0.090; 95% CI, 0.057–0.123; DeLong *p* < 0.001). The difference between APACHE II and SOFA was not statistically significant (ΔAUC, 0.020; 95% CI, −0.009–0.049; *p* = 0.178) (Table 6, Figure 3).

Pairwise comparisons of the correlated ROC curves are presented in Table 7. The AUC of the simplified RIN-IM Score was 0.890 (95% CI, 0.861–0.919), compared with 0.820 (95% CI, 0.788–0.852) for APACHE II and 0.800 (95% CI, 0.766–0.834) for SOFA. The absolute AUC differences were 0.070 (95% CI, 0.038–0.102; DeLong *p* < 0.001) and 0.090 (95% CI, 0.057–0.123; DeLong *p* < 0.001), respectively. The AUC difference between APACHE II and SOFA was 0.020 (95% CI, −0.009 to 0.049; *p* = 0.178). Addition of the RIN-IM Score to the clinical reference model increased the apparent AUC from 0.821 to 0.923 (ΔAUC, 0.102; 95% CI, 0.078–0.126; DeLong *p* < 0.001). The nested-model likelihood-ratio test yielded χ^2^ = 166.4 (*p* < 0.001). The optimism-corrected AUC increased from 0.810 to 0.912, while the calibration slope changed from 0.90 to 0.93 and the calibration intercept from −0.04 to −0.02. The Brier score decreased from 0.160 to 0.112 (Table 7).

**Figure 4:**
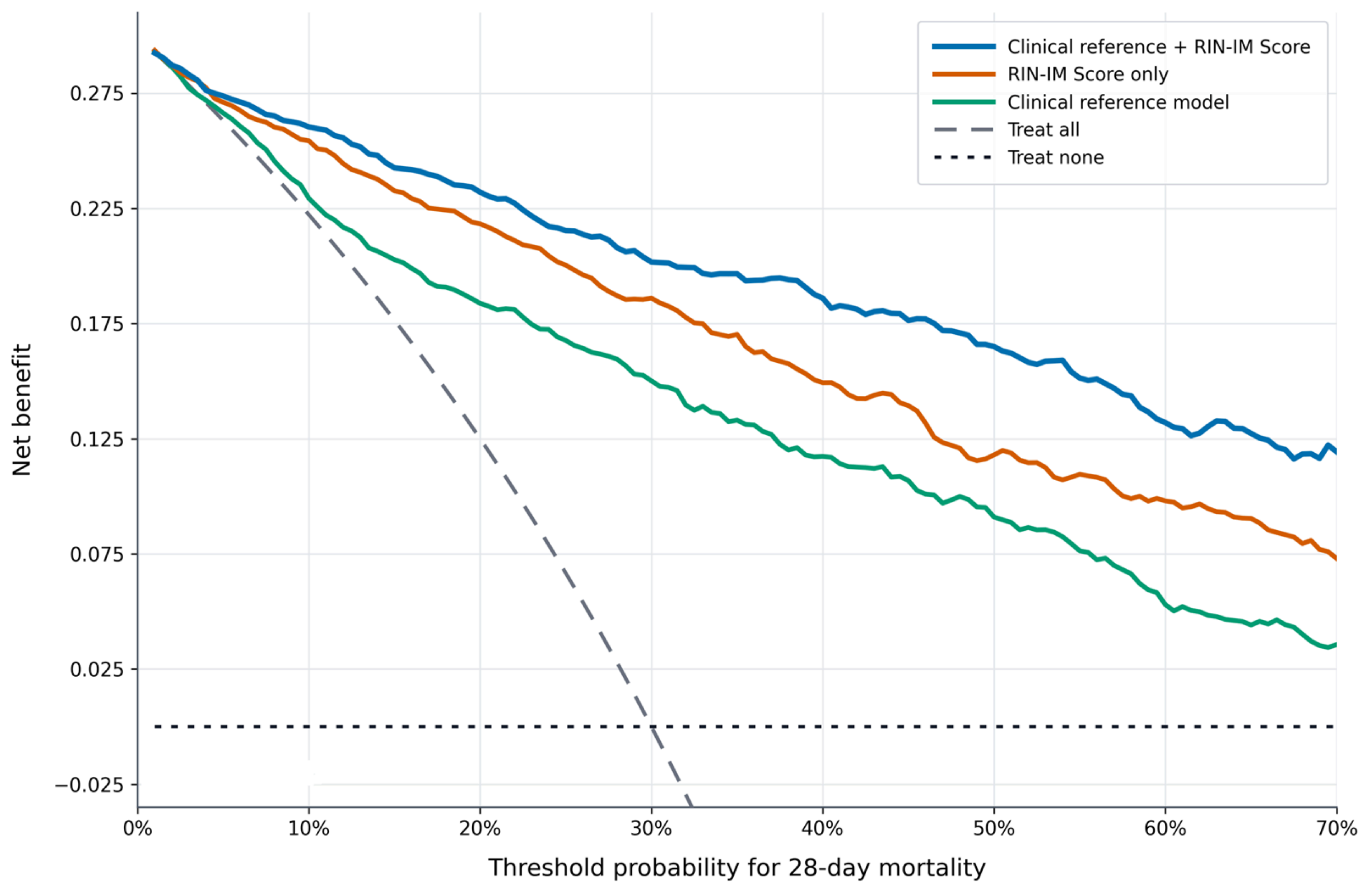
Decision-curve analysis for prediction of 28-day mortality.

Decision-curve analysis showed that the clinical reference model plus the RIN-IM Score had the highest net benefit across threshold probabilities from 10% to 60% (Figure 4). At a 10% threshold, the net-benefit values were 0.260 for the combined model, 0.254 for the RIN-IM Score-only model, 0.229 for the clinical reference model, and 0.222 for the treat-all strategy. The corresponding values at thresholds of 20%, 30%, 40%, 50%, and 60% were 0.232, 0.202, 0.186, 0.165, and 0.132 for the combined model; 0.218, 0.186, 0.149, 0.118, and 0.098 for the RIN-IM Score-only model; and 0.184, 0.150, 0.117, 0.091, and 0.053 for the clinical reference model, respectively. At the 30% threshold, the absolute net-benefit gain of the combined model was 0.052 relative to the clinical reference model and 0.016 relative to the RIN-IM Score-only model, corresponding to approximately 5.2 and 1.6 additional net true-positive classifications per 100 patients, respectively. At the 50% threshold, the corresponding gains were 0.074 and 0.047, equivalent to 7.4 and 4.7 additional net true-positive classifications per 100 patients. The treat-none strategy had a net benefit of zero at all thresholds (Figure 4).

**Figure 5:**
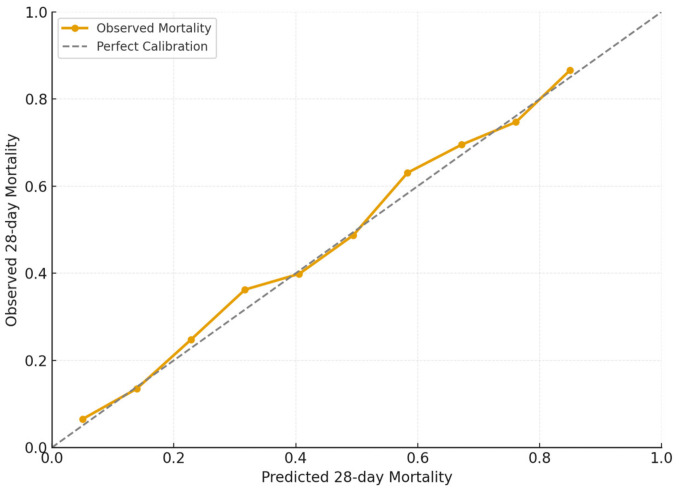
Calibration plot illustrating the agreement between predicted and observed 28-day mortality across RIN-IM Score deciles.

The calibration plot showing the agreement between predicted and observed 28-day mortality is presented in Figure 5. Predicted mortality values increased gradually from approximately 0.05 in the lowest decile to 0.85 in the highest decile. Observed mortality followed a closely aligned pattern, ranging from roughly 0.06 in the lowest risk group to 0.88 in the highest group. The calibration curve closely tracked the 45-degree reference line, particularly in the mid-range of predicted risk (0.30–0.70), where observed mortality differed by less than ±0.05 from the ideal line, indicating excellent model fit (Figure 5).

### 3.6. Sensitivity and Subgroup Analyses

The median interval from ICU admission to completion of all RIN-IM component sampling was 2.05 h (IQR, 1.10–4.25 h). All required components were collected within 2 h in 476 patients (47.6%) and within 6 h in 842 patients (84.2%). Complete blood count and biochemical specimens originated from the same phlebotomy episode in 438 patients (43.8%). Sampling was completed before all reliably timestamped major interventions in 592 patients (59.2%), after at least one intervention in 318 patients (31.8%), and had indeterminate temporal status in 90 patients (9.0%). In the ≤2 h sensitivity cohort, the apparent and optimism-corrected AUCs were 0.886 and 0.872, respectively, compared with 0.890 and 0.878 in the complete cohort. In the pre-intervention subset, the corresponding AUCs were 0.883 and 0.870. The complete numerical results are presented in Supplementary Table S4.

Cox proportional-hazards sensitivity analyses are presented in Supplementary Table S5. Each one-point increase in the RIN-IM Score was associated with an unadjusted hazard ratio of 1.63 (95% CI, 1.55–1.71; *p* < 0.001) and an adjusted hazard ratio of 1.46 (95% CI, 1.38–1.54; *p* < 0.001). Compared with the low-risk group, the adjusted hazard ratios were 2.94 (95% CI, 1.97–4.39; *p* < 0.001) for the intermediate-risk group and 9.28 (95% CI, 6.22–13.85; *p* < 0.001) for the high-risk group. Addition of the continuous RIN-IM Score to the clinical reference model increased Harrell’s C-index from 0.795 to 0.891 and yielded a likelihood-ratio χ^2^ of 151.8 (*p* < 0.001). The global Schoenfeld test was non-significant for all models (all *p* > 0.05) (Supplementary Table S5). Kaplan–Meier curves demonstrated progressive separation across the low-, intermediate-, and high-risk categories (log-rank *p* < 0.001; Supplementary Figure S1).

Exploratory subgroup performance is presented in Supplementary Table S6. The simplified RIN-IM Score yielded AUCs of 0.889 (95% CI, 0.857–0.921) in medical admissions and 0.881 (95% CI, 0.819–0.943) in surgical admissions. The corresponding AUCs were 0.874 (95% CI, 0.829–0.919) in patients with sepsis or septic shock and 0.895 (95% CI, 0.861–0.929) in non-sepsis admissions. Across diagnostic subgroups with at least 20 deaths, the simplified-score AUC ranged from 0.869 to 0.896, calibration slopes ranged from 0.84 to 0.93, and Brier scores ranged from 0.119 to 0.171. The omnibus interaction *p* values were 0.638 for primary admission diagnosis and 0.574 for calendar period. Performance measures were not estimated for the trauma, postoperative, gastrointestinal or hepatic, and renal, metabolic, or toxicological subgroups because each contained fewer than 20 deaths (Supplementary Table S6).

After additional adjustment for calendar period and COVID-19 diagnosis, each one-point increase in the RIN-IM Score was associated with higher odds of 28-day mortality (adjusted OR, 1.65; 95% CI, 1.51–1.81; *p* < 0.001). The optimism-corrected AUC was 0.913, the calibration slope was 0.94, the calibration intercept was −0.02, and the Brier score was 0.111.

## 4. Discussion

The present study evaluated a novel composite biomarker-based score, the RIN-IM Score, for predicting 28-day mortality in a heterogeneous ICU population and demonstrated that this index provides strong and independent prognostic information beyond established severity scores such as APACHE II and SOFA. In a cohort of 1000 critically ill adults, RIN-IM not only showed a clear stepwise increase in mortality across low-, intermediate-, and high-risk categories (7.6%, 27.2%, and 77.3%, respectively), but also achieved higher discriminative performance (AUC 0.89) compared with APACHE II (AUC 0.82) and SOFA (AUC 0.80). Each 1-point increase in the RIN-IM Score was associated with a 48% rise in the odds of 28-day mortality, even after adjustment for age, comorbidity burden, APACHE II, SOFA, and major clinical interventions. These findings suggest that integrating renal, inflammatory, nutritional, and metabolic information into a single score captures dimensions of risk that are only partially reflected by traditional physiology-based scoring systems.

The rationale for evaluating RIN-IM is not that APACHE II, SOFA, or bedside clinical assessment are unnecessary. Nor is RIN-IM intended to determine ICU admission, replace clinical judgment, or provide additional value in every patient whose prognosis is already clinically apparent. ICU admission may depend on diagnosis, anticipated trajectory, monitoring requirements, local staffing, and procedural considerations that are not represented by any biomarker score. The proposed role of RIN-IM is narrower: to provide a standardized laboratory-based estimate of mortality risk and to test whether this estimate adds prognostic information to an established clinical model. In the present cohort, addition of RIN-IM improved discrimination, calibration, overall prediction error, and decision-curve net benefit. However, these statistical improvements do not establish that routinely calculating the score changes clinical decisions or improves outcomes. Clinical context remains essential, and external validation and impact studies are required to determine whether RIN-IM has a useful role in practice.

The present cohort represented a heterogeneous ICU population comprising medical, surgical, septic, respiratory, neurological, cardiovascular, trauma, and postoperative admissions. Sepsis or septic shock was the most frequent primary diagnosis, although substantial proportions of patients were admitted for other acute conditions. The distribution of admission diagnoses differed between survivors and non-survivors, emphasizing the influence of diagnostic spectrum on baseline mortality risk. Exploratory subgroup analyses showed some variation in discrimination and calibration across admission categories; however, the interaction tests for admission type, sepsis status, primary diagnosis, and calendar period were not statistically significant. These findings should not be interpreted as demonstrating equivalent performance across all subgroups because several diagnostic categories contained limited numbers of deaths and yielded wide confidence intervals. Differences in case mix may affect both baseline risk and the distributions of the biomarkers incorporated into the RIN-IM Score.

Vincent et al. originally developed the SOFA score to quantify organ dysfunction in sepsis, and subsequent work has confirmed its prognostic value across various ICU populations [2]. Tian et al. reported that dynamic changes in APACHE II improved outcome prediction compared with baseline values alone, highlighting that severity scores remain useful but imperfect tools for outcome prediction [3]. Beigmohammadi et al. and Wang et al. also showed that both APACHE II and SOFA are independently associated with mortality in COVID-19 and acute kidney injury cohorts, but their AUCs typically range between 0.70 and 0.85 in contemporary studies [4,5]. Shahi et al. and Kumar et al. further demonstrated that while these scores are helpful, their performance varies across diagnostic groups, particularly in neurological and mixed medical–surgical ICU populations [6,7]. Li et al. found that combining SOFA with simple laboratory markers improved risk stratification in sepsis patients [8]. In this context, our finding that the RIN-IM Score outperforms APACHE II and SOFA, while using only routine laboratory parameters obtained within the first 24 h, indicates that biologically anchored indices may refine risk assessment beyond complex physiology-based models.

Previous studies have associated the individual RIN-IM components with adverse outcomes across oncological, cardiovascular, metabolic, and critical-care populations [9,10,11,12,13,14,15,16,17,18,19,20,21,28,29,30,31,32,33,34,35,36,37,38,39,40,41,42]. However, much of the evidence concerning PIV and TyG originates from non-ICU cohorts. Differences in case mix, fasting status, treatment exposure, and biomarker distributions limit direct extrapolation of those findings to critically ill patients. ICU-specific studies provide support for the prognostic relevance of inflammatory cell-count indices, albumin, and renal dysfunction, but they do not validate the present combination, thresholds, or point assignments. The previous literature therefore supports the biological and prognostic rationale for evaluating these predictors, whereas the RIN-IM model and its simplified scoring algorithm remain newly developed and require external validation.

Regarding the inflammatory component of the score, Yang et al. demonstrated in a large meta-analysis that the SII is a powerful predictor of survival in several malignancies, emphasizing the prognostic importance of neutrophil–lymphocyte–platelet interactions [12]. Fois et al. showed that SII on admission predicted in-hospital mortality in patients with COVID-19 pneumonia, supporting its value in acute systemic inflammation [11]. Uçar et al. extended these observations to hematologic malignancies, reporting that SII and related indices were independently associated with overall survival [13]. Wang et al. reported that blood count–derived inflammatory markers, including SII, were correlated with sepsis-associated delirium in older adults, underlining their relevance across diverse ICU syndromes [14]. Our results are consistent with these findings: both SII and PIV were significantly higher in non-survivors and remained independent predictors of mortality in multivariable analysis, with each 1000-unit increase in SII and each 500-unit increase in PIV conferring 12% and 19% higher mortality risk, respectively. Bilgin et al. previously showed in pulmonary thromboembolism that PIV and TyG are strong predictors of mortality, and in a separate acute coronary syndrome cohort that PIV, TyG, and triglyceride/HDL ratio were associated with mortality and diagnostic differentiation [10,16]. Our study extends these observations to a general ICU population and integrates both SII and PIV into a composite score rather than evaluating each index in isolation.

The TyG index was originally developed using fasting triglyceride and glucose measurements as a surrogate marker of insulin resistance in metabolically stable populations [44]. Momani et al. reported a robust association between TyG and cardiovascular disease in patients with type 2 diabetes mellitus, while Yakout et al. identified a link between TyG and vitamin D status in postmenopausal women, suggesting broader metabolic implications [19,20]. Bilgin et al. showed that TyG was associated with mortality and thrombus burden in ST-elevation myocardial infarction [21]. In the present study, fasting status could not be reliably verified because the cohort comprised unselected patients admitted to the ICU. The calculated index should therefore be regarded as an admission-based TyG measure reflecting the combined effects of dysglycemia and hypertriglyceridemia during early critical illness rather than as a direct measure of chronic insulin resistance. Non-survivors had higher admission-based TyG values, and the index remained associated with 28-day mortality in the continuous biomarker model. However, acute stress, recent feeding, intravenous glucose, insulin, corticosteroids, catecholamines, and nutritional support may influence both components of the index. Accordingly, the thresholds derived in this cohort may not be directly comparable with fasting TyG thresholds reported in ambulatory or cardiovascular populations.

Atherogenic lipid patterns captured by the AIP have also been linked to adverse outcomes. Dobiásová first described AIP as a logarithmic transformation of the triglyceride/HDL ratio, correlating with lipoprotein particle size and atherogenic potential [17]. Lan et al. and Zhou et al. showed that higher AIP levels were associated with worse clinical outcomes and physical dysfunction in peritoneal dialysis patients and older adults, respectively [22,23]. Lian et al. and Li et al. further demonstrated that AIP was related to both incident cardiovascular disease and the severity of coronary artery stenosis, while Wu et al. and Wang et al. linked AIP to coronary artery disease in postmenopausal women and incident diabetes [24,25,26,27]. Although AIP itself did not enter our final multivariable model, non-survivors had significantly higher AIP values than survivors, supporting the broader concept that pro-atherogenic and pro-inflammatory lipid profiles contribute to mortality risk in ICU patients.

The nutritional and renal components of the RIN-IM Score are supported by a substantial body of evidence. Huang et al. showed that albumin-corrected anion gap was associated with mortality in ICU patients with heart failure and acute kidney injury, indirectly emphasizing albumin’s modifying role in risk assessment [28]. Bai et al. and Turcato et al. reported that hypoalbuminemia in sepsis and sepsis-induced coagulopathy was associated with increased mortality and transfusion requirements [29,30]. Wu et al. provided mechanistic insight by linking hypoalbuminemia in COVID-19 to pulmonary capillary leakage [31]. Eygi and Bayrakci showed that the albumin-to-D-dimer ratio predicted mortality in mechanically ventilated ICU patients, while Khor et al. and Xiao et al. demonstrated that protein-energy wasting and albumin-related ratios independently predicted adverse outcomes in acute kidney injury and sepsis [32,33,34]. Li et al. also highlighted the prognostic value of an inflammation-based index that incorporates albumin in sepsis-associated AKI [35]. Higher serum albumin concentrations were inversely associated with 28-day mortality; however, this observational association should not be interpreted as evidence of a causal protective effect.

Lin et al. evaluated the serum albumin-to-creatinine ratio in 9690 patients with sepsis from the multicenter eICU database and reported a non-linear inverse association with 28-day ICU mortality [36]. Wang et al. similarly reported that a lower serum-derived albumin-to-creatinine ratio was associated with all-cause mortality among ICU patients with heart failure [37]. In the present cohort, sAlb/Cr was lower among non-survivors than among survivors. However, this finding was treated as exploratory because the ratio is mathematically determined by serum albumin and serum creatinine, is dependent on the laboratory units used in its calculation, and was not evaluated as an independent component of the RIN-IM Score. Importantly, sAlb/Cr is biologically and analytically distinct from urinary albumin-to-creatinine ratio and should not be interpreted as a measure of albuminuria or glomerular albumin leakage.

Our study also aligns with and extends previous work focusing on composite inflammation–nutrition indices. Li et al. recently reported that the advanced lung cancer inflammation index (ALI), a composite of albumin, BMI, and neutrophil–lymphocyte ratio, predicted 28-day mortality in sepsis-associated acute kidney injury, supporting the concept that multi-domain indices out-perform single markers [35]. Segmen et al. proposed vitamin D, GRP, and inflammatory markers as a combined prognostic panel in ICU patients, again highlighting the value of integrating pathways rather than examining single biomarkers in isolation [15]. Compared with these approaches, the RIN-IM Score is distinctive in several respects: it is built entirely from routinely available laboratory parameters; it simultaneously incorporates renal, inflammatory, nutritional, and insulin–metabolic axes; and it was benchmarked directly against APACHE II and SOFA with higher discriminative calibration. To our knowledge, no previous study has proposed an ICU mortality score that jointly integrates SII, PIV, TyG, albumin, and creatinine in this manner.

The observed risk gradients should not be interpreted as validated clinical-action thresholds. In particular, the 7.6% mortality observed in the low-risk category is not sufficiently low to justify step-down transfer or de-escalation based on the RIN-IM Score alone. The present study did not evaluate score-guided clinical decisions, and ICU disposition should continue to incorporate diagnosis, physiological trajectory, treatment requirements, monitoring needs, and clinician judgment.

For practical implementation, RIN-IM would require automatic calculation from laboratory data and integration into the electronic health record or ICU clinical information system. Its potential advantage over more complex machine-learning or artificial-intelligence models is not greater predictive sophistication, but parsimony, transparency, reproducibility, and reliance on a small number of routinely measured variables. Conversely, RIN-IM is a static, single-time-point model and cannot capture continuously changing physiological signals, treatment responses, or complex interactions that dynamic AI-based systems may incorporate. No head-to-head comparison with an AI-based deterioration or mortality model was performed; therefore, superiority over such systems cannot be claimed. Future studies should evaluate automated implementation, usability, alert burden, temporal recalibration, and comparative performance against validated dynamic prediction models.

This study has several strengths. It included a relatively large and clinically heterogeneous ICU cohort encompassing major medical and surgical admission categories, permitting evaluation across a broad case mix within a tertiary-care setting. The score was derived entirely from routinely available laboratory measurements obtained during a predefined early admission window, and the potential influence of sampling time was examined in early-sampling sensitivity analyses. Model evaluation included assessment of continuous predictor–outcome relationships, bootstrap internal validation of the complete development process, calibration assessment, formal comparison of correlated AUCs, incremental-value testing, decision-curve analysis, and prespecified sensitivity and subgroup analyses. In addition, the RIN-IM components have clinical interpretability, reflecting renal dysfunction, systemic immune-inflammatory activation, albumin-related alterations during acute illness, and metabolic stress.

These findings should be interpreted within a prognostic-prediction framework. The observed associations indicate that the RIN-IM components and total score were related to 28-day mortality, whereas discrimination and calibration describe the model’s ability to estimate and stratify mortality risk. Neither these associations nor the observed predictive performance establish that the component biomarkers causally contribute to mortality or that interventions modifying their values would improve clinical outcomes.

### Limitations

This study has several limitations. Its retrospective, single-center design may have introduced selection bias and limits transportability to ICUs with different case mixes, referral patterns, laboratory practices, and treatment protocols. The complete-case approach may also have introduced bias if patients with missing measurements differed systematically from those included. Residual confounding by unmeasured factors, including premorbid frailty, pre-ICU treatment, detailed therapeutic strategies, and changes in organ function during the ICU stay, cannot be excluded. Moreover, each RIN-IM component was represented by a single early measurement. Although the first available values within 24 h of ICU admission were used and early-sampling sensitivity analyses were performed, the components were not invariably obtained immediately upon admission or from the same phlebotomy episode. Some measurements may therefore have been influenced by fluid resuscitation, vasopressors, corticosteroids, insulin or intravenous glucose, nutritional support, transfusion, mechanical ventilation, or renal replacement therapy. Interventions initiated before ICU admission and treatments without reliable timestamps could not be fully characterized. Fasting status also could not be consistently verified; consequently, use of the fasting-derived TyG index with admission glucose and triglyceride measurements in critically ill patients requires cautious interpretation.

The present model used only the first available measurements and therefore represents baseline prognostic assessment rather than dynamic risk monitoring. Creatinine, albumin, inflammatory indices, glucose, and triglycerides may change substantially during critical illness in response to disease progression and treatment. Serial RIN-IM measurements or biomarker trajectories may provide information beyond a single admission value and should be evaluated using appropriately designed longitudinal or time-dependent prediction models.

Stable preadmission creatinine, cumulative fluid administration before laboratory sampling, arterial pH, and a validated premorbid frailty measure were not consistently available. Consequently, admission creatinine could not be interpreted relative to individual baseline renal function, and the potential effects of plasma-volume expansion and acid–base status on albumin concentrations could not be fully evaluated. Palliative-care consultation, repeat operations, open body cavities, massive-transfusion status, cause-specific mortality, and decisions to withhold or withdraw life-sustaining treatment were not systematically adjudicated. These unmeasured clinical factors may have influenced both biomarker values and mortality. Traumatic brain injury was not separately classified within the available trauma and neurological categories; therefore, TBI-specific performance could not be evaluated. Patients with class III obesity were uncommon in the present cohort, comprising only 24 of 1000 patients (2.4%). Because severe obesity may influence inflammatory, metabolic, and renal biomarkers, the discrimination and calibration of the RIN-IM models in patients with a BMI ≥ 40 kg/m^2^ remain uncertain. This limited representation should be considered when assessing the generalizability of our findings to severely obese ICU populations.

Although the complete model-development process was internally validated using bootstrap resampling, internal validation cannot establish performance in independent populations. The thresholds used for the simplified RIN-IM Score were derived at least partly from the present cohort and may be sample-dependent. Categorization of continuous predictors can cause information loss, threshold instability, and reduced transportability; accordingly, the continuous model was considered primary and the point-based score a secondary pragmatic simplification. The proposed thresholds, point assignments, and risk categories require external validation before clinical implementation. The study period also encompassed the COVID-19 pandemic and associated changes in admission patterns, treatment strategies, and resource availability, potentially introducing temporal heterogeneity. Although calendar-period and diagnostic-subgroup analyses were performed, some subgroups contained few outcome events and were not sufficiently powered for definitive comparisons. Finally, the study evaluated prognostic performance for 28-day all-cause mortality but did not determine whether score-guided clinical decisions improve patient outcomes. Future studies should externally validate the model across institutions, healthcare periods, and diagnostic groups and investigate long-term survival, functional status, quality of life, and organ-specific outcomes.

## 5. Conclusions

In conclusion, in this retrospective single-center ICU cohort, the continuous RIN-IM model showed good discrimination and calibration for predicting 28-day mortality. The simplified 0–10 point score, derived from routinely available early laboratory measurements reflecting renal dysfunction, systemic immune-inflammatory activation, albumin-related alterations, and metabolic stress, retained most of the predictive information of the continuous model. The RIN-IM Score demonstrated higher apparent discrimination than APACHE II and SOFA and provided incremental prognostic information when added to the clinical reference model. However, these findings are based on internal validation and should not be interpreted as evidence of clinical superiority or utility. Prospective external validation and clinical-impact studies are required before the score can be recommended for routine risk stratification or treatment decision-making.

## Figures and Tables

**Table 1:** Complete calculation algorithm and derivation of the simplified RIN-IM Score.

Domain	Component	0 Points	1 Point	2 Points	Possible Domain Score
Renal	Serum creatinine (mg/dL)	<1.50	1.50–1.99	≥2.00	0–2
Inflammatory	SII	<1000	1000–2999	≥3000	0–4 *
Inflammatory	PIV	<500	500–1199	≥1200	0–4 *
Nutritional/protein status	Serum albumin (g/dL)	≥3.50	2.50–3.49	<2.50	0–2
Insulin–metabolic	TyG index	<9.00	9.00–9.49	≥9.50	0–2
Total RIN-IM Score					0–10

* The inflammatory domain includes both SII and PIV; therefore, its combined possible range is 0–4 points. SII = neutrophil count × platelet count/lymphocyte count; PIV = neutrophil count × monocyte count × platelet count/lymphocyte count; TyG index = ln[triglycerides (mg/dL) × admission glucose (mg/dL)/2]. Complete blood count components were expressed in ×10^9^/L. The total RIN-IM Score was calculated by summing the five component scores. Low-, intermediate-, and high-risk categories were defined as 0–3, 4–6, and 7–10 points, respectively.

**Table 2:** Demographic characteristics, admission severity measures, and early clinical interventions according to 28-day survival status.

Variable	Total (*N* = 1000)	Survivors (*n* = 700)	Non-Survivors (*n* = 300)	*p* Value
	Mean ± SD or *n* (%) or Median, IQR			
Age, years	63.4 ± 15.2	60.1 ± 14.7	70.1 ± 14.3	0.001
Gender, Male	580 (58%)	400 (57.1%)	180 (60.0%)	0.370
BMI, kg/m^2^	27.6 ± 5.1	27.9 ± 5.0	27.0 ± 5.3	0.040
Hypertension	540 (54%)	350 (50%)	190 (63.3%)	0.012
Diabetes mellitus	380 (38%)	235 (33.6%)	145 (48.3%)	0.011
Coronary artery disease	260 (26%)	150 (21.4%)	110 (36.7%)	0.014
Pre-existing CKD	150 (15%)	70 (10%)	80 (26.7%)	0.016
**Admission type**				**<0.001**
Medical admission	760 (76.0%)	510 (72.9%)	250 (83.3%)	
Surgical admission, including trauma	240 (24.0%)	190 (27.1%)	50 (16.7%)	
**Primary ICU admission diagnosis**				**<0.001**
Sepsis or septic shock	310 (31.0%)	170 (24.3%)	140 (46.7%)	
Acute respiratory failure, non-COVID-19	170 (17.0%)	125 (17.9%)	45 (15.0%)	
Acute neurological disease	120 (12.0%)	92 (13.1%)	28 (9.3%)	
Cardiovascular disease	95 (9.5%)	73 (10.4%)	22 (7.3%)	
COVID-19-related critical illness	90 (9.0%)	60 (8.6%)	30 (10.0%)	
Postoperative admission	80 (8.0%)	70 (10.0%)	10 (3.3%)	
Trauma	75 (7.5%)	63 (9.0%)	12 (4.0%)	
Gastrointestinal or hepatic disease	35 (3.5%)	27 (3.9%)	8 (2.7%)	
Renal, metabolic, or toxicological disease	25 (2.5%)	20 (2.9%)	5 (1.7%)	
APACHE II score	19.8 ± 7.6	17.3 ± 6.2	25.2 ± 6.8	0.024
SOFA score at admission	7.8 ± 3.5	6.5 ± 2.8	10.6 ± 3.4	0.025
Mechanical ventilation (first 24 h)	520 (52%)	290 (41.4%)	230 (76.7%)	0.001
Vasopressor use (first 24 h)	340 (34%)	150 (21.4%)	190 (63.3%)	0.001
ICU length of stay, days	8 (4–14)	7 (4–12)	10 (5–17)	0.016

Data are presented as mean ± standard deviation, median (interquartile range), or number (column percentage), as appropriate. Admission type and primary ICU admission diagnosis were classified separately. Primary admission diagnoses were mutually exclusive and represented the immediate indication for ICU admission. In patients with multiple coexisting conditions, the principal diagnosis documented in the ICU admission record was used. Medical admissions were defined as admissions without a preceding operative or primarily trauma-related indication. Surgical admissions included scheduled or emergency postoperative admissions and trauma patients managed by a surgical service. The *p* values represent omnibus comparisons of the overall distributions between survivors and non-survivors and were calculated using the chi-square test; separate *p* values were not calculated for individual diagnostic categories.

**Table 3:** Comparison of inflammatory, nutritional, renal, and metabolic biomarkers between survivors and non-survivors.

Biomarker/Index	Survivors (*n* = 700)	Non-Survivors (*n* = 300)	*p* Value
	Mean ± SD or Median (IQR)		
Neutrophil count (×10^9^/L)	8.2 ± 3.5	11.4 ± 4.8	0.003
Lymphocyte count (×10^9^/L)	1.25 ± 0.46	0.82 ± 0.34	0.002
Platelet count (×10^9^/L)	240 ± 85	188 ± 70	0.009
Monocyte count (×10^9^/L)	0.58 ± 0.22	0.74 ± 0.25	0.014
CRP (mg/L)	85 (40–132)	148 (96–220)	0.021
SII (Systemic Immune-Inflammation Index)	1600 (900–2500)	3800 (2500–5800)	0.001
PIV (Pan-Immune-Inflammation Value)	650 (320–980)	1680 (980–2550)	0.002
Albumin (g/dL)	3.2 ± 0.6	2.6 ± 0.5	0.004
Serum albumin-to-creatinine ratio (sAlb/Cr), g/mg	2.8 ± 1.0	1.7 ± 0.9	0.006
Serum creatinine (mg/dL)	1.18 ± 0.46	1.92 ± 0.72	0.030
AIP (Atherogenic Index of Plasma)	0.28 ± 0.22	0.41 ± 0.25	0.023
Admission triglycerides (mg/dL)	136 ± 52	162 ± 68	0.048
Admission glucose (mg/dL)	146 ± 45	182 ± 62	0.031
Admission-based TyG index	8.85 ± 0.52	9.32 ± 0.58	0.012
RIN-IM Score (0–10)	3.2 ± 1.4	7.2 ± 1.6	0.001

**Table 4:** Separate multivariable models for 28-day mortality.

Model and Predictor	Effect Specification		OR (95% CI)		*p* Value
**Model A: Clinical reference model**					
Age	Per 10-year increase		1.22 (1.10–1.35)		<0.001
Male sex	Male vs. female		1.09 (0.82–1.45)		0.554
Hypertension	Present vs. absent		1.14 (0.86–1.51)		0.365
Diabetes mellitus	Present vs. absent		1.32 (1.00–1.75)		0.049
Coronary artery disease	Present vs. absent		1.21 (0.89–1.64)		0.221
Chronic kidney disease	Present vs. absent		1.56 (1.11–2.20)		0.011
SOFA score	Per 1-point increase		1.24 (1.18–1.30)		<0.001
**Model B: Biomarker-component model**					
Serum creatinine	75th vs. 25th percentile		1.68 (1.39–2.04)		<0.001
Serum albumin	75th vs. 25th percentile		0.58 (0.48–0.70)		<0.001
ln(SII)	75th vs. 25th percentile		1.54 (1.28–1.84)		<0.001
ln(PIV)	75th vs. 25th percentile		1.46 (1.22–1.75)		<0.001
TyG index	75th vs. 25th percentile		1.61 (1.31–1.98)		<0.001
**Model C: RIN-IM Score-only model**					
RIN-IM Score	Per 1-point increase		1.88 (1.75–2.02)		<0.001
**Model D: Clinical reference model plus RIN-IM Score**					
Age	Per 10-year increase		1.12 (1.00–1.26)		0.054
Male sex	Male vs. female		1.03 (0.75–1.41)		0.862
Hypertension	Present vs. absent		1.01 (0.74–1.38)		0.948
Diabetes mellitus	Present vs. absent		1.18 (0.89–1.58)		0.250
Coronary artery disease	Present vs. absent		1.12 (0.81–1.56)		0.493
Chronic kidney disease	Present vs. absent		1.29 (0.91–1.84)		0.153
SOFA score	Per 1-point increase		1.14 (1.08–1.21)		<0.001
RIN-IM Score	Per 1-point increase		1.68 (1.54–1.84)		<0.001
**Model performance**	**Apparent AUC** **(95% CI)**	**Optimism-** **Corrected AUC**	**Calibration** **Slope**	**Calibration** **Intercept**	**Brier** **Score**
Model A: Clinical reference model	0.821 (0.793–0.849)	0.810	0.90	−0.04	0.160
Model B: Biomarker-component model	0.907 (0.885–0.929)	0.898	0.94	−0.02	0.123
Model C: RIN-IM Score-only model	0.890 (0.861–0.919)	0.878	0.91	−0.04	0.132
Model D: Clinical reference model plus RIN-IM Score	0.923 (0.904–0.942)	0.912	0.93	−0.02	0.112

OR, odds ratio; CI, confidence interval; AUC, area under the receiver operating characteristic curve; SOFA, Sequential Organ Failure Assessment; SII, systemic immune-inflammation index; PIV, pan-immune-inflammation value; TyG, triglyceride-glucose index. SII and PIV were natural-log transformed before analysis. In Model B, effects are expressed as odds ratios comparing the 75th with the 25th percentile of each continuous predictor. For predictors modeled using restricted cubic splines, these contrasts were calculated from the fitted spline function. The total RIN-IM Score and its constituent biomarkers were not included in the same model. Calibration measures and Brier scores are optimism-corrected estimates obtained from 1000 bootstrap resamples. APACHE II and SOFA were not entered simultaneously; analyses replacing SOFA with APACHE II are presented in Supplementary Table S3.

**Table 5:** Distribution of RIN-IM Score categories (low-, intermediate-, high-risk) and associated mortality rates.

RIN-IM Risk Category	Score Range	Number of Patients (*n*)	28-Day Mortality (*n*, %)	Mortality Rate (%)
Low Risk	0–3	420	32 (7.6%)	7.6%
Intermediate Risk	4–6	360	98 (27.2%)	27.2%
High Risk	≥7	220	170 (77.3%)	77.3%
Total	—	1000	300 (30%)	30%

**Table 6:** Discriminative performance, optimal cut-offs, and paired DeLong comparisons for prediction of 28-day mortality. Panel (**A**): Discriminative performance and data-derived cut-offs. Panel (**B**): Pairwise comparisons of correlated AUCs.

**(A)**					
**Model or Score**	**Optimal Cut-Off**	**AUC (95% CI)**	**Sensitivity, %**	**Specificity, %**	**Youden Index**
RIN-IM Score	≥6	0.890 (0.861–0.919)	84.0	81.3	0.653
APACHE II	≥21	0.820 (0.788–0.852)	71.2	74.0	0.452
SOFA	≥8	0.800 (0.766–0.834)	68.5	72.1	0.406
**(B)**					
**Paired Comparison**		**AUC 1** **(95% CI)**	**AUC 2** **(95% CI)**	**Absolute ΔAUC** **(95% CI)**	**DeLong** ***p*** **Value**
RIN-IM Score vs. APACHE II		0.890 (0.861–0.919)	0.820 (0.788–0.852)	0.070 (0.038–0.102)	<0.001
RIN-IM Score vs. SOFA		0.890 (0.861–0.919)	0.800 (0.766–0.834)	0.090 (0.057–0.123)	<0.001
APACHE II vs. SOFA		0.820 (0.788–0.852)	0.800 (0.766–0.834)	0.020 (−0.009–0.049)	0.178
Continuous RIN-IM model vs. simplified RIN-IM Score		0.907 (0.885–0.929)	0.890 (0.861–0.919)	0.017 (0.007–0.028)	0.001
Clinical reference model plus RIN-IM Score vs. clinical reference model		0.923 (0.904–0.942)	0.821 (0.793–0.849)	0.102 (0.078–0.126)	<0.001

APACHE II, Acute Physiology and Chronic Health Evaluation II; AUC, area under the receiver operating characteristic curve; CI, confidence interval; RIN-IM, Renal–Inflammation–Nutrition–Insulin/Metabolism; SOFA, Sequential Organ Failure Assessment.

**Table 7:** Pairwise comparisons of discrimination and incremental prognostic performance.

Comparison	AUC 1 (95% CI)	AUC 2 (95% CI)	Absolute ΔAUC (95% CI)	*p* Value
RIN-IM Score vs. APACHE II	0.890 (0.861–0.919)	0.820 (0.788–0.852)	0.070 (0.038–0.102)	<0.001
RIN-IM Score vs. SOFA	0.890 (0.861–0.919)	0.800 (0.766–0.834)	0.090 (0.057–0.123)	<0.001
APACHE II vs. SOFA	0.820 (0.788–0.852)	0.800 (0.766–0.834)	0.020 (−0.009–0.049)	0.178
Continuous RIN-IM model vs. simplified RIN-IM Score	0.907 (0.885–0.929)	0.890 (0.861–0.919)	0.017 (0.007–0.028)	0.001
Clinical reference model plus RIN-IM vs. clinical reference model	0.923 (0.904–0.942)	0.821 (0.793–0.849)	0.102 (0.078–0.126)	<0.001

AUC, area under the receiver operating characteristic curve; CI, confidence interval; APACHE II, Acute Physiology and Chronic Health Evaluation II; SOFA, Sequential Organ Failure Assessment. Absolute differences in correlated AUCs were evaluated using paired DeLong tests. Positive ΔAUC values favor the first model listed in each comparison. The clinical reference model included age, sex, prespecified comorbidities, and SOFA.

## Data Availability

The original contributions presented in this study are included in the article/Supplementary Material. Further inquiries can be directed to the corresponding author.

## Supplementary Materials

The following supporting information can be downloaded at: https://www.mdpi.com/article/10.3390/diagnostics16152443/s1, Table S1. Functional forms and adjusted associations of the continuous RIN-IM predictors with 28-day mortality. Table S2. Correlations and collinearity diagnostics for variables evaluated in the RIN-IM models. Table S3. Sensitivity analyses replacing SOFA with APACHE II in the clinical reference models for 28-day mortality. Table S4. Timing of RIN-IM component sampling and early-sampling sensitivity analyses. Table S5. Cox proportional-hazards sensitivity analyses for 28-day mortality. Table S6. Performance of the RIN-IM models across admission diagnoses and clinically relevant subgroups. Figure S1. Kaplan–Meier survival curves for 28-day mortality according to RIN-IM risk categories.

## Abbreviations

sAlb/Cr	Serum albumin-to-creatinine ratio
uACR	Urinary albumin-to-creatinine ratio
AIP	Atherogenic Index of Plasma
APACHE II	Acute Physiology and Chronic Health Evaluation II
aOR	Adjusted Odds Ratio
AUC	Area Under the Curve
BMI	Body Mass Index
CI	Confidence Interval
CRP	C-reactive Protein
ICU	Intensive Care Unit
OR	Odds Ratio
PIV	Pan-Immune-Inflammation Value
ROC	Receiver Operating Characteristic
RIN-IM	Renal–Inflammation–Nutrition–Insulin/Metabolism Score
SD	Standard Deviation
SII	Systemic Immune-Inflammation Index
SOFA	Sequential Organ Failure Assessment
TyG	Triglyceride-Glucose Index
