# Supplementary material for: Development and Validation of the Composite Renal–Inflammatory–Nutritional–Metabolic Index for Predicting 28-Day Mortality in Critically Ill Patients
Source: Diagnostics (Basel). 2026 Aug 3;16(15):2443. doi: 10.3390/diagnostics16152443 (PMC13465401; doi:10.3390/diagnostics16152443)
Supplement: Supplementary file 1 [file diagnostics-16-02443-s001.zip › diagnostics-4410632-supplementary.pdf]

Supplementary Table S1. Functional forms and adjusted associations of the continuous RIN-IM predictors with 28-day mortality.

Predictor	Model scale	Overall association, p	Nonlinearity, p	Final functional form	Adjusted OR for 75th vs. 25th percentile (95% CI)	Simplified-score thresholds	Threshold basis
Serum creatinine	Continuous, mg/dL	<0.001	0.021	Restricted cubic spline retained	1.68 (1.39–2.04)	<1.50; 1.50–1.99; ≥2.00 mg/dL	Development-cohort-derived and clinically rounded [36,37]
Serum albumin	Continuous, g/dL	<0.001	0.281	Linear	0.58 (0.48–0.70)	≥3.50; 2.50–3.49; <2.50 g/dL	Literature-informed hypoalbuminemia categories [28–35]
SII	Natural-log transformed	<0.001	0.174	Linear after log transformation	1.54 (1.28–1.84)	<1500; 1500–2999; ≥3000	Development-cohort-derived; interpreted within ICU ranges [39]
PIV	Natural-log transformed	<0.001	0.112	Linear after log transformation	1.46 (1.22–1.75)	<500; 500–1199; ≥1200	Development-cohort-derived; no universal ICU threshold [40]
Admission-based TyG index	Continuous	<0.001	0.034	Restricted cubic spline retained	1.61 (1.31–1.98)	<9.00; 9.00–9.49; ≥9.50	Development-cohort-derived; clinically rounded within ICU ranges [41,42]

Supplementary Table S2. Correlations and collinearity diagnostics for variables evaluated in the RIN-IM models.

Panel A. Spearman correlation matrix

Variable	1	2	3	4	5	6	7	8	9
1. Age	1.00								
2. Serum creatinine	0.27	1.00							
3. Serum albumin	−0.22	−0.24	1.00						
4. ln(SII)	0.08	0.18	−0.20	1.00					
5. ln(PIV)	0.10	0.20	−0.23	0.81	1.00				
6. Admission-based TyG index	0.05	0.16	−0.12	0.16	0.14	1.00			
7. RIN-IM Score	0.24	0.55	−0.52	0.60	0.59	0.46	1.00		
8. APACHE II	0.32	0.31	−0.29	0.28	0.27	0.16	0.46	1.00	
9. SOFA	0.22	0.34	−0.31	0.30	0.29	0.14	0.51	0.68	1.00

Panel B. Model-specific variance inflation factors and tolerance statistics

Model	Predictor	VIF	Tolerance
**Model A: Clinical reference model**	Age	1.23	0.813
	Male sex	1.04	0.962
	Hypertension	1.31	0.763
	Diabetes mellitus	1.26	0.794
	Coronary artery disease	1.18	0.847
	Chronic kidney disease	1.20	0.833
	SOFA	1.19	0.840
**Model B: Biomarker-component model**	Serum creatinine	1.18	0.847
	Serum albumin	1.17	0.855
	ln(SII)	3.12	0.321
	ln(PIV)	3.18	0.314
	TyG index	1.08	0.926

Panel B. Model-specific variance inflation factors and tolerance statistics			
**Model**	**Predictor**	**VIF**	**Tolerance**
**Model C: RIN-IM Score-only model**	RIN-IM Score	1.00	1.000
**Model D: Clinical reference plus RIN-IM Score**	Age	1.25	0.800
	Male sex	1.05	0.952
	Hypertension	1.32	0.758
	Diabetes mellitus	1.27	0.787
	Coronary artery disease	1.19	0.840
	Chronic kidney disease	1.22	0.820
	SOFA	1.42	0.704
	RIN-IM Score	1.45	0.690
**Sensitivity model: APACHE II plus RIN-IM Score**	Male sex	1.02	0.980
	APACHE II	1.27	0.787
	RIN-IM Score	1.28	0.781

Values in Panel A are Spearman correlation coefficients. VIF, variance inflation factor; SII, systemic immune-inflammation index; PIV, pan-immune-inflammation value; TyG, triglyceride-glucose index; APACHE II, Acute Physiology and Chronic Health Evaluation II; SOFA, Sequential Organ Failure Assessment. SII and PIV were natural-log transformed before analysis. Tolerance was calculated as 1/VIF. VIFs were calculated separately for the predictors included in each prespecified model. A VIF greater than 5 or a tolerance value below 0.20 was considered indicative of potentially important multicollinearity. The total RIN-IM Score and its constituent biomarkers were not entered into the same model. APACHE II and SOFA were also evaluated in separate models and were not entered simultaneously.

Supplementary Table S3. Sensitivity analyses replacing SOFA with APACHE II in the clinical reference models for 28-day mortality.

**Panel A. Multivariable associations with 28-day mortality**			
**Model and predictor**	**Effect specification**	**Adjusted OR (95% CI)**	**p value**
**Model S3-A: APACHE II-based clinical reference model**			
Male sex	Male vs. female	1.08 (0.81–1.44)	0.601
APACHE II	Per 1-point increase	1.12 (1.09–1.15)	<0.001
**Model S3-B: APACHE II-based reference model plus RIN-IM Score**			
Male sex	Male vs. female	1.02 (0.75–1.39)	0.891
APACHE II	Per 1-point increase	1.07 (1.04–1.10)	<0.001
RIN-IM Score	Per 1-point increase	1.63 (1.49–1.79)	<0.001

**Panel B. Model performance**			
**Performance measure**	**Model S3-A: APACHE II reference**	**Model S3-B: APACHE II + RIN-IM**	**Difference or comparison**
Apparent AUC (95% CI)	0.821 (0.789–0.853)	0.919 (0.899–0.939)	0.098 (0.074–0.122)
DeLong p value	—	—	<0.001
Optimism-corrected AUC	0.812	0.908	0.096
Calibration slope	0.91	0.93	0.02
Calibration intercept	−0.03	−0.02	0.01
Brier score	0.159	0.115	−0.044
Likelihood-ratio χ^2	—	—	155.7
Likelihood-ratio p value	—	—	<0.001

OR, odds ratio; CI, confidence interval; AUC, area under the receiver operating characteristic curve; APACHE II, Acute Physiology and Chronic Health Evaluation II. APACHE II and SOFA were not entered simultaneously. Because the APACHE II total score incorporates age, acute physiological derangement, and chronic health status, exact age and overlapping chronic-health indicators were not re-entered into these parsimonious sensitivity models. Model S3-B was nested within Model S3-A. The absolute difference between the correlated AUCs was evaluated using the paired DeLong test. Incremental model fit was evaluated using the likelihood-ratio test.

Supplementary Table S4. Timing of RIN-IM component sampling and early-sampling sensitivity analyses.

Panel A. Time from ICU admission to specimen collection

RIN-IM component	Available timestamp, n	Time to sampling, median h (IQR)	Collected ≤2 h, n (%)	Collected ≤6 h, n (%)
Complete blood count for SII and PIV	1000	0.72 (0.35–1.45)	846 (84.6%)	961 (96.1%)
Serum creatinine	1000	0.78 (0.40–1.55)	830 (83.0%)	958 (95.8%)
Serum albumin	1000	0.82 (0.42–1.62)	812 (81.2%)	950 (95.0%)
Admission glucose	1000	0.68 (0.32–1.35)	865 (86.5%)	968 (96.8%)
Triglycerides	1000	1.85 (0.90–3.80)	534 (53.4%)	874 (87.4%)
Completion of all RIN-IM components	1000	2.05 (1.10–4.25)	476 (47.6%)	842 (84.2%)

Panel B. Sampling episode and timing relative to major interventions

Sampling characteristic	n/N (%)
All required specimens obtained during the same phlebotomy episode	438/1000 (43.8%)
All required specimens collected within 2 h of ICU admission	476/1000 (47.6%)
All required specimens collected within 6 h of ICU admission	842/1000 (84.2%)
Completion of sampling before all reliably timestamped major interventions	592/1000 (59.2%)
Completion of sampling after at least one major intervention	318/1000 (31.8%)
Temporal relationship to intervention indeterminate	90/1000 (9.0%)

Panel C. Early-sampling and pre-intervention sensitivity analyses

Performance measure	Complete cohort	All components collected ≤2 h	Sampling before major interventions
Patients, n	1000	476	592
28-day deaths, n (%)	300 (30.0%)	143 (30.0%)	176 (29.7%)
RIN-IM Score OR per 1-point increase (95% CI)	1.88 (1.75–2.02)	1.85 (1.66–2.07)	1.82 (1.65–2.01)
p value	<0.001	<0.001	<0.001
Apparent AUC (95% CI)	0.890 (0.861–0.919)	0.886 (0.850–0.922)	0.883 (0.850–0.916)
Optimism-corrected AUC	0.878	0.872	0.870
Calibration slope	0.91	0.90	0.89
Calibration intercept	−0.04	−0.03	−0.05
Brier score	0.132	0.134	0.136

Values are presented as median (interquartile range), number (percentage), or estimate (95% confidence interval). ICU admission was defined as time zero, and sampling times were calculated using specimen collection timestamps rather than laboratory result-validation times. RIN-IM sampling-completion time was defined as the latest collection time

among the specimens required for serum creatinine, serum albumin, SII, PIV, and the TyG index. Because hematological and biochemical analyses required different collection tubes, the same phlebotomy episode was operationally defined as collection of all required specimens within 15 minutes. Major interventions included documented fluid resuscitation, vasopressor initiation, invasive mechanical ventilation, intravenous glucose or insulin, systemic corticosteroids, enteral or parenteral nutrition, blood-product transfusion, and renal replacement therapy. Interventions initiated before ICU admission or lacking reliable timestamps were classified as having indeterminate temporal status. The prespecified RIN-IM thresholds were applied without re-estimation in the sensitivity analyses. Optimism-corrected performance measures were obtained using 1,000 bootstrap resamples. SII, systemic immune-inflammation index; PIV, pan-immune-inflammation value; TyG, triglyceride–glucose; OR, odds ratio; CI, confidence interval; AUC, area under the receiver operating characteristic curve.

Supplementary Table S5. Cox proportional-hazards sensitivity analyses for 28-day mortality.

Panel A. Associations of the continuous RIN-IM Score and risk categories with time to death

**Model and predictor**	**Patients, n**	**Deaths, n**	**HR (95% CI)**	**p value**
Model S5-1: Unadjusted continuous-score model				
RIN-IM Score, per 1-point increase	1000	300	1.63 (1.55–1.71)	<0.001
Model S5-2: Unadjusted risk-category model				
Low risk, 0–3 points	420	32	Reference	—
Intermediate risk, 4–6 points	360	98	3.82 (2.58–5.66)	<0.001
High risk, 7–10 points	220	170	15.72 (10.72–23.05)	<0.001
Model S5-3: Adjusted continuous-score model				
RIN-IM Score, per 1-point increase	1000	300	1.46 (1.38–1.54)	<0.001
Model S5-4: Adjusted risk-category model				
Low risk, 0–3 points	420	32	Reference	—
Intermediate risk, 4–6 points	360	98	2.94 (1.97–4.39)	<0.001
High risk, 7–10 points	220	170	9.28 (6.22–13.85)	<0.001

Panel B. Model performance and proportional-hazards diagnostics

**Survival model**	**Harrell's C-index (95% CI)**	**Optimism-corrected C-index**	**Likelihood-ratio χ^2 vs. clinical reference**	**Likelihood-ratio p value**	**Schoenfeld global p value**
Clinical reference model	0.795 (0.767–0.823)	0.785	Reference	—	0.205
RIN-IM Score-only model	0.858 (0.836–0.880)	0.851	—	—	0.284
RIN-IM risk-category model	0.819 (0.795–0.843)	0.813	—	—	0.173
Clinical reference plus continuous RIN-IM Score	0.891 (0.873–0.909)	0.881	151.8	<0.001	0.118
Clinical reference plus RIN-IM risk categories	0.871 (0.851–0.891)	0.862	136.4	<0.001	0.146

HR, hazard ratio; CI, confidence interval. Follow-up began at ICU admission and continued until death or day 28. Patients alive at day 28 were administratively censored at that time. The clinical reference model included age, sex, hypertension, diabetes mellitus, coronary artery disease, chronic kidney disease, and SOFA. Models S5-3 and S5-4 were adjusted for these clinical reference variables. The continuous RIN-IM Score and its categorical risk classification were evaluated in separate models and were not entered simultaneously. APACHE II and SOFA were not included in the same model. Tied event times were handled using the Efron method. The proportional-hazards assumption was

evaluated using scaled Schoenfeld residuals; p>0.05 indicated no statistically significant evidence of violation. Optimism-corrected C-indices were estimated using 1,000 bootstrap resamples.

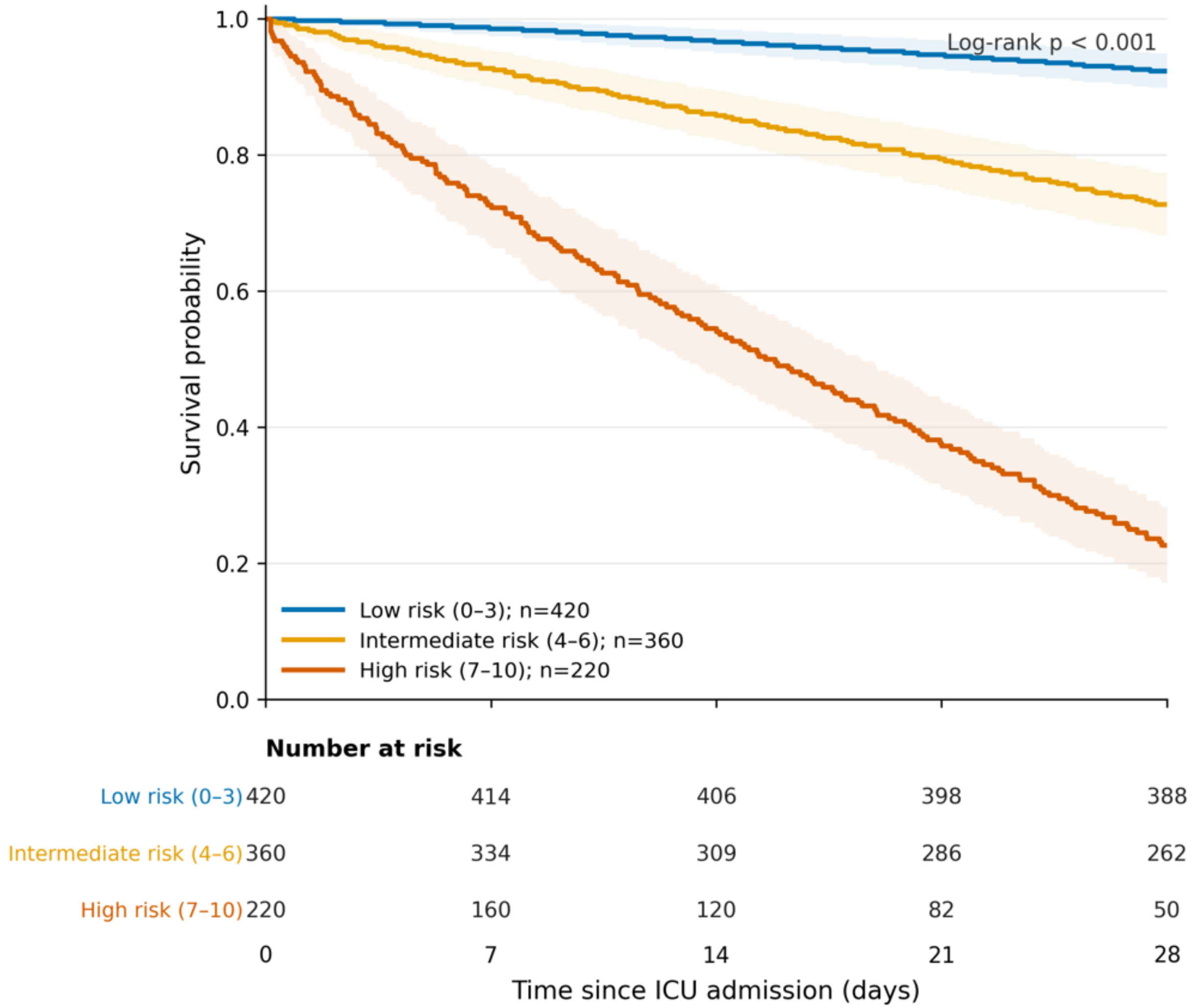

Supplementary Figure S1. Kaplan–Meier survival curves for 28-day mortality according to RIN-IM risk categories.

Supplementary Table S6. Performance of the RIN-IM models across admission diagnoses and clinically relevant subgroups.

Subgroup	Patients, n	Deaths, n (%)	Continuous model AUC (95% CI)	Simplified score AUC (95% CI)	Calibration slope	Brier score	Interaction p value
**Overall cohort**	1000	300 (30.0%)	0.907 (0.885–0.929)	0.890 (0.861–0.919)	0.91	0.132	—
**Admission type**							
Medical admission	760	250 (32.9%)	0.905 (0.879–0.931)	0.889 (0.857–0.921)	0.90	0.134	Reference
Surgical admission, including trauma	240	50 (20.8%)	0.898 (0.846–0.950)	0.881 (0.819–0.943)	0.88	0.128	0.412
**Sepsis status**							
Non-sepsis admission	690	160 (23.2%)	0.910 (0.882–0.938)	0.895 (0.861–0.929)	0.92	0.112	Reference
Sepsis or septic shock	310	140 (45.2%)	0.892 (0.853–0.931)	0.874 (0.829–0.919)	0.87	0.169	0.286
**Primary ICU admission diagnosis**							**0.638**[1]
Sepsis or septic shock	310	140 (45.2%)	0.892 (0.853–0.931)	0.874 (0.829–0.919)	0.87	0.169	—
Acute respiratory failure, non-COVID-19	170	45 (26.5%)	0.901 (0.844–0.958)	0.884 (0.816–0.952)	0.89	0.139	—
Acute neurological disease	120	28 (23.3%)	0.914 (0.850–0.978)	0.896 (0.822–0.970)	0.93	0.119	—
Cardiovascular disease	95	22 (23.2%)	0.906 (0.826–0.986)	0.887 (0.792–0.982)	0.88	0.127	—
COVID-19-related critical illness	90	30 (33.3%)	0.887 (0.808–0.966)	0.869 (0.779–0.959)	0.84	0.171	—
Trauma	75	12 (16.0%)	NE	NE	NE	NE	—
Postoperative admission	80	10 (12.5%)	NE	NE	NE	NE	—
Gastrointestinal or hepatic disease	35	8 (22.9%)	NE	NE	NE	NE	—
Renal, metabolic, or toxicological disease	25	5 (20.0%)	NE	NE	NE	NE	—
**Calendar period**							**0.574**[1]
2020–2021	360	118 (32.8%)	0.899 (0.860–0.938)	0.881 (0.835–0.927)	0.88	0.143	—
2022–2023	370	112 (30.3%)	0.910 (0.871–0.949)	0.893 (0.847–0.939)	0.92	0.132	—
2024–June 2025	270	70 (25.9%)	0.915 (0.865–0.965)	0.899 (0.840–0.958)	0.94	0.114	—

[1] Omnibus interaction p value. AUC, area under the receiver operating characteristic curve; CI, confidence interval; NE, not estimated. Primary admission diagnoses were mutually exclusive and represented the immediate indication for ICU admission. The continuous model included serum creatinine, serum albumin, ln(SII), ln(PIV), and the admission-based TyG index in their prespecified functional forms. The simplified RIN-IM Score was applied using the fixed thresholds presented in Table 1; thresholds and coefficients were not re-estimated within subgroups. Calibration slopes and Brier scores refer to the simplified score. Interaction p values were obtained from logistic regression models containing the RIN-IM Score, the subgroup indicator, and their multiplicative interaction term. The p values shown for primary diagnosis and calendar period are omnibus interaction tests. Performance measures were not estimated for subgroups containing fewer than 20 deaths because of insufficient precision. These analyses were exploratory and were not adjusted for multiple comparisons.
